# Structure of SpoT reveals evolutionary tuning of catalysis via conformational constraint

**DOI:** 10.1038/s41589-022-01198-x

**Published:** 2022-12-05

**Authors:** Hedvig Tamman, Karin Ernits, Mohammad Roghanian, Andres Ainelo, Christina Julius, Anthony Perrier, Ariel Talavera, Hanna Ainelo, Rémy Dugauquier, Safia Zedek, Aurelien Thureau, Javier Pérez, Gipsi Lima-Mendez, Régis Hallez, Gemma C. Atkinson, Vasili Hauryliuk, Abel Garcia-Pino

**Affiliations:** 1 Cellular and Molecular Microbiology, Faculté des Sciences, Université libre de Bruxelles (ULB), Boulevard du Triomphe, Brussels, Belgium; 2 Department of Experimental Medicine, University of Lund, Lund, Sweden; 3 Department of Chemistry, Umeå University, Umeå, Sweden; 4 Department of Molecular Biology, Umeå University, Umeå, Sweden; 5 Departement of Clinical Microbiology, Rigshospitalet, Copenhagen, Denmark; 6 Biology of Microorganisms Research Unit, Namur Research Institute for Life Science, University of Namur, Namur, Belgium; 7 Bacterial Cell Cycle and Development, Biology of Microorganisms Research Unit, Namur Research Institute for Life Science, University of Namur, Namur, Belgium; 8 Synchrotron SOLEIL, Saint-Aubin - BP 48, Gif sur Yvette, France; 9 WELBIO, Brussels, Belgium; 10 University of Tartu, Institute of Technology, Tartu, Estonia

**Keywords:** X-ray crystallography, Biophysical chemistry, Bacteria, Translation, Enzyme mechanisms

## Abstract

Stringent factors orchestrate bacterial cell reprogramming through increasing the level of the alarmones (p)ppGpp. In Beta- and Gammaproteobacteria, SpoT hydrolyzes (p)ppGpp to counteract the synthetase activity of RelA. However, structural information about how SpoT controls the levels of (p)ppGpp is missing. Here we present the crystal structure of the hydrolase-only SpoT from *Acinetobacter baumannii* and uncover the mechanism of intramolecular regulation of ‘long’-stringent factors. In contrast to ribosome-associated Rel/RelA that adopt an elongated structure, SpoT assumes a compact τ-shaped structure in which the regulatory domains wrap around a Core subdomain that controls the conformational state of the enzyme. The Core is key to the specialization of long RelA-SpoT homologs toward either synthesis or hydrolysis: the short and structured Core of SpoT stabilizes the τ-state priming the hydrolase domain for (p)ppGpp hydrolysis, whereas the longer, more dynamic Core domain of RelA destabilizes the τ-state priming the monofunctional RelA for efficient (p)ppGpp synthesis.

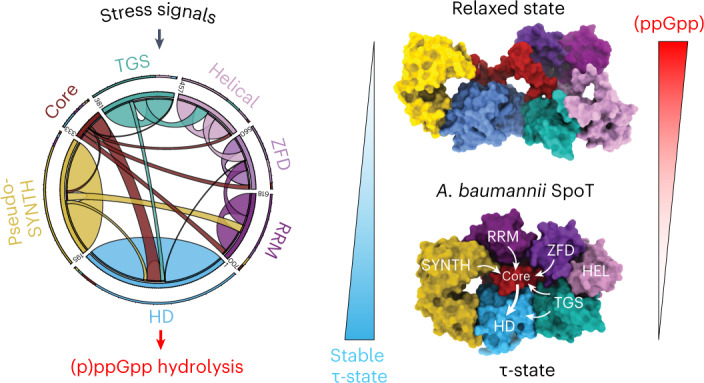

## Abstract (web summary)

The structure of SpoT assumes a compact τ-shaped structure in which the regulatory domains wrap around a core subdomain that controls the conformational state of the enzyme and primes it for (p)ppGpp hydrolysis.

## Main

Long RelA-SpoT homolog (RSH) stringent factors regulate virtually all aspects of bacterial physiology by controlling the levels of the signaling nucleotide alarmones guanosine pentaphosphate and tetraphosphate, collectively referred to as (p)ppGpp^1–6^. The ribosome-associated RSH RelA is a dedicated amino acid starvation sensor with a strong (p)ppGpp synthesis activity (SYNTH) that is induced on detection of deacylated tRNA in the ribosomal A site^7,8^ and no detectable hydrolase (HD) activity^9^. The SYNTH activity of RelA is balanced by SpoT, a bifunctional RSH with a strong, Mn^2+^-dependent HD activity^10,11^ and weak SYNTH activity^12^. The RelA-SpoT pair is a product of gene duplication of the ancestral bifunctional ribosome-associated RSH Rel^1^.

**Fig. 1:**
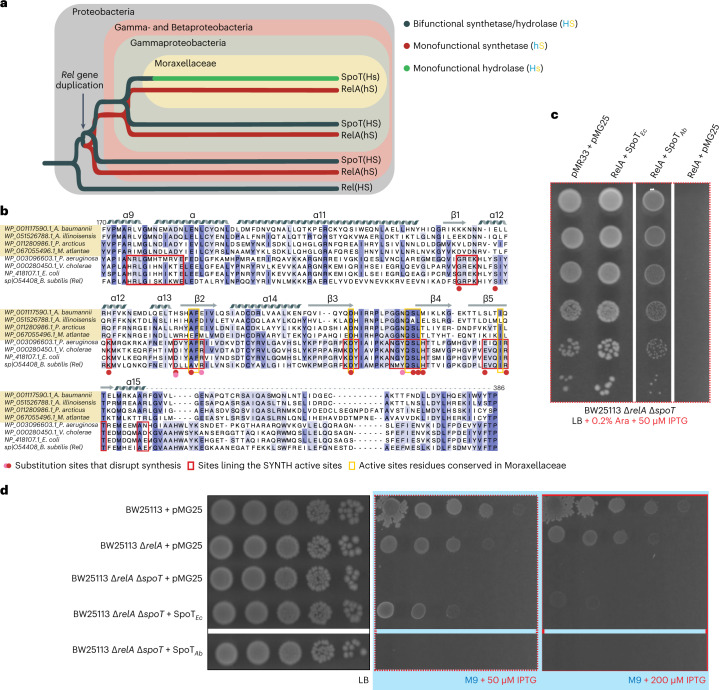
*A. baumannii* SpoT is a monofunctional alarmone HD. **a**, Evolution of long RSHs in Proteobacteria. Duplication of the ancestral bifunctional RSH Rel in Beta- and Gammaproteobacterial lineages gave rise to RelA and SpoT, leading to subfunctionalization of RelA as monofunctional SYNTH-only alarmone synthetase and SpoT as a predominantly HD RSH. In the Moraxellaceae family of Gammaproteobacteria, SpoT has undergone further subfunctionalization, evolving into a monofunctional HD-only alarmone HD. **b**, Alignment of SYNTH-critical regions in long RSHs highlights the sequence divergence in Moraxellaceae SpoTs. **c**, Coexpression of SpoT_*Ab*_ counteracts the growth defect in ppGpp^0^ (Δ*relA* Δ*spoT*) *E. coli* caused by RelA expression. This demonstrates that SpoT_*Ab*_ is HD active in the *E. coli* host. **d**, While the SYNTH activity of ectopically expressed SpoT_*Ec*_ is essential and sufficient for promoting the growth of ppGpp^0^ *E. coli* on M9 minimal medium, SpoT_*Ab*_ fails to promote the growth of Δ*relA* Δ*spoT E. coli* on M9. This demonstrates that, unlike SpoT_*Ec*_, which is SYNTH-active, SpoT_*Ab*_ is SYNTH-inactive.

Subfunctionalization—the partitioning of functions between two paralogues that arose through gene duplication—appears to have happened at least twice in Gammaproteobacterial long RSHs (Fig. 1a). First, relatively soon after the duplication that gave rise to *relA* and *spoT*, RelA evolved into a monofunctional, SYNTH-only RSH. Second, during the evolution of the Moraxellaceae lineage, SpoT likely lost its synthetase function (Fig. 1a,b)^1^. This resulted in further specialization into monofunctional (p)ppGpp hydrolase, SpoT[Hs] (uppercase ‘H’ for HD-competent and lowercase ‘s’ indicates ‘synthetase-incompetent’), as opposed to the bifunctional HD- and SYNTH-competent SpoT[HS] found in other Beta- and Gammaproteobacteria. Recent studies of the Moraxellaceae *A. baumannii* indicate a lack of (p)ppGpp in a Δ*relA* strain, both with and without acute amino acid starvation induced by serine hydroxamate^13,14^, suggesting that RelA is the sole source of alarmones. Furthermore, consistent with the key role of (p)ppGpp in bacterial virulence and antibiotic tolerance^15,16^, the *A. baumannii* Δ*relA* strain displays increased sensitivity to multiple antibiotics^13,14^ and decreased virulence^14^.

While the physiological role of SpoT as a virulence and stress tolerance factor is well established^17,18^, structural studies of SpoT have so far been unsuccessful. Here we provide the long-missing structural insight into the molecular mechanism of SpoT. We show that *A. baumannii* SpoT (SpoT_*Ab*_) is a monofunctional (p)ppGpp HD and uncover how its C-terminal domain (CTD) is an allosteric activator of the HD function. The structure of the full-length SpoT_*Ab*_ complexed with ppGpp reveals a compact conformation in which all the regulatory domains wrap around a Core domain that connects the pseudo-SYNTH and TGS (ThrRS, GTPase and SpoT) domains. This Core is one of the intrinsically disordered regions (IDR) present in Rel and RelA when in the active synthetase state. In SpoT_*Ab*_, Core and TGS cooperate to align and activate the HD-active site while translating allosteric feedback from the CTD to modulate the HD output. Finally, we propose a unifying conceptual framework that rationalizes the relative balance between HD versus SYNTH activities of long RSHs Rel, RelA and SpoT, finetuned through the entropic force produced by IDRs that function as conformational gatekeepers of the enzyme.

## Results

### *A. baumannii* SpoT_*Ab*_ is a monofunctional HD long RSH

The loss of residues critical for SYNTH activity suggests that Moraxellaceae SpoT enzymes underwent subfunctionalization to become long RSH hydrolases (Fig. 1a,b). Similar to RelA’s pseudo-HD domain, the SYNTH domain has been retained in Moraxellaceae SpoT as a presumably noncatalytic pseudo-SYNTH, suggesting its involvement in stabilization or allosteric regulation of the HD domain. To probe the hydrolysis function of *A. baumannii* SpoT (SpoT_*Ab*_) in live cells, we leveraged SpoT_*Ab*_’s hydrolysis being crucial for controlling the cellular levels of (p)ppGpp, making *spoT* conditionally essential in the *relA*^+^ *Escherichia coli*^12^. We cotransformed a ppGpp^0^ (Δ*relA*/Δ*spoT*) *E. coli* strain with (1) a pMG25-based plasmid driving the isopropyl-β-d-thiogalactoside- (IPTG-)inducible expression of *spoT*_Ab_ under the control of P_A1/O4/O3_ and (2) a pMR33 derivative for arabinose-inducible expression of *relA*_Ec_ under the control of P_BAD_. While expression of the (p)ppGpp synthetase RelA_*Ec*_ strongly inhibited the growth of ppGpp^0^ *E. coli*, ectopic coexpression of SpoT_*Ab*_ restored growth completely (Fig. 1c and Supplementary Data), demonstrating that SpoT_*Ab*_ is HD active in the surrogate *E. coli* host.

Next, we used our dual plasmid coexpression system to probe the SYNTH activity of SpoT RSHs. ppGpp^0^ *E. coli* is auxotrophic for 11 amino acids, and (p)ppGpp synthetase activity of SpoT_*Ec*_ is essential for growth of Δ*relA E. coli* on minimal medium^12^. Unlike the SYNTH-active SpoT_*Ec*_, SpoT_*Ab*_ failed to promote the growth of ppGpp^0^ *E. coli* on M9 minimal medium (Fig. 1d), confirming that SpoT_*Ab*_ is SYNTH-inactive. Taken together, these results demonstrate that SpoT_*Ab*_ is a monofunctional long RSH hydrolase.

### SpoT_*Ab*_ has a compact mushroom-like τ-shaped structure

**Fig. 2:**
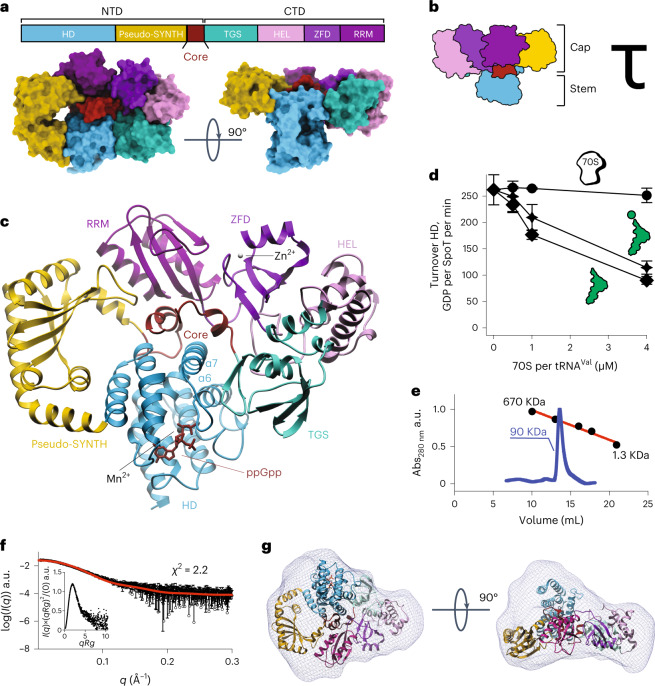
Full-length monomeric *A. baumannii* SpoT adopts a compact ‘mushroom’-shaped HD-active τ-state. **a**, Structure of ‘mushroom’-shaped SpoT_*Ab*_–ppGpp complex in the τ-state. The domain organization, from N to C terminus: NTDs HD, pseudo-synthetase (pseudo-SYNTH) and Core domains, and CTDs, TGS, HEL, ZFD and RRM. The ppGpp alarmone is in red. **b**, Cartoon representation the SpoT_*Ab*_. The ‘stem’ of the mushroom is formed by the enzymatic HD domain and the ‘cap’ by the regulatory domains: NTD pseudo-SYNTH domain and the CTD. **c**, Ribbon representation of the SpoT_*Ab*_–ppGpp complex. The α6/α7 motif is held in the hydrolysis-compatible position by the folded Core domain and the TGS β-hairpin, with the Core domain communicating allosteric signals to HD from the regulatory domains. **d**, The HD activity of SpoT_*Ab*_ is insensitive to the addition of *E. coli* 70S ribosomes, and nonspecifically weakly inhibited by both aminoacylated and deacyated *E. coli* tRNA^Val^. **e**, Analytical SEC of SpoT_*Ab*_ supports its monomeric nature in solution. a.u., arbitrary units. **f**, Experimental SAXS analysis of SpoT_*Ab*_ at 8 mg ml^−1^ further confirms the monomeric nature of SpoT_*Ab*_. The analysis of the normalized Kratky plot (insert) of the SAXS curve reveals folded globular shape of SpoT_*Ab*_. **g**, Ab initio envelope of SpoT_*Ab*_ reconstructed from the experimental SAXS data superimposed on the crystal structure. Comparison of both models shows that in solution the enzyme adopts the same conformation as observed in the crystal. Error bars represent a s.d. of three or more independent samples examined over three independent experiments.

The 2.5 Å resolution X-ray structure of full-length catalytically active SpoT_*Ab*_ bound to ppGpp revealed a multidomain architecture different from that of ribosome-bound long RSHs Rel and RelA^19–22^ (Fig. 2a–c and Supplementary Table 1). The HD, pseudo-SYNTH, TGS, helical (HEL), Zn-finger (ZFD) and RNA recognition motif (RRM) domains of SpoT_*Ab*_ form a mushroom-like tau (τ)-shaped quaternary structure (Fig. 2a–c). In this arrangement, pseudo-SYNTH, TGS, HEL, ZFD and RRM all lie in a single plane and form a compact disk-like structure that constitutes the ‘cap’ of the ‘mushroom’ (Fig. 2b). A helix-turn-helix domain (residues 334 to 379) that provides the transition between the N-terminal domain (NTD) and CTD regions, lies at the ‘Core’ of the ‘cap’ and seemingly mediates interactions among all domains of the enzyme. Such an arrangement suggests that this Core domain—disordered in Rel/RelA structures—stabilizes the disk-like ‘cap’ of SpoT (Fig. 2c). Moreover, the Core provides the HD domain with a physical link to all the other domains of SpoT_*Ab*_. Finally, the HD protrudes from the plane of the ‘cap’ in the opposite direction of the C-terminal RRM domain, forming the ‘stem’ of the mushroom (Fig. 2b,c).

In the τ-state SYNTH and TGS are sequestered in the ‘cap’ contrasting with ribosome-bound Rel and RelA in which it directly inspects the deacylated transfer RNA CCA-3′ end in the A site^20–23^. While we do detect a mild inhibitory effect of tRNA on SpoT_*Ab*_ hydrolysis, the effect is insensitive to tRNA aminoacylation status (Fig. 2d). This nonspecific inhibition contrast with the HD activity of bifunctional SpoT_*Ec*_, exclusively inhibited by deacylated tRNA^24^. Finally, the ZFD and RRM are held in place by the Core via contact sites that in Rel/RelA mediate ribosomal RNA recognition^19–23^, suggesting that in the τ-conformation, SpoT_*Ab*_ should be incompatible with ribosome recruitment. In good agreement with this structural prediction, while ribosomes strongly suppress the HD activity of *Bacillus subtilis* Rel (Rel_*Bs*_)^25^, the addition of *E. coli* 70S ribosomes has no effect on the activity of SpoT_*Ab*_ (Fig. 2d). Thus, our biochemical results indicate that while SpoT_*Ab*_ can likely associate with RNA in a nonspecific way, it is a ribosome-independent enzyme.

### Shorter IDRs correlate with SpoT HD specialization

The presence of IDRs at the α6–α7 loop^26^, the Core domain, and the linker between HEL/ZFD domains in RelA and Rel (Supplementary Fig. 1a) has posed an experimental challenge for structural studies^19–22^ of long-RSH enzymes. The molecular function of these flexible regions, unresolved in the structures, is unknown. Comparison between SpoT_*Ab*_ and ribosome-bound RelA/Rel suggests the unfolding of Core and HEL domains is part of the conformational switch that positions TGS, ZFD and RRM domains to stimulate the synthesis activity of Rel/RelA on ribosome recruitment (Supplementary Fig. 1b).

The length of these disordered or flexible regions is on average shorter in monofunctional SpoT and much longer in the monofunctional RelA. Bifunctional Rels have interdomain IDRs of sizes between both monofunctional enzymes (Supplementary Table 2). The α6–α7 loop of the HD domain of SpoT[Hs] is three times shorter than that of RelA, which, in turn, is twice longer than that of bifunctional Rel (Supplementary Table 2). The same pattern is observed for the other two IDRs: the Core domain and the region connecting HEL and ZFD domains. This is consistent with the significantly lower propensity for disorder of the Core of SpoT_*Ab*_ compared to RelA_*Ab*_ (Supplementary Fig. 1c,d). We speculate that these IDRs have evolved to stabilize either τ- (shorter IDRs) or elongated (longer IDRs) states of monofunctional SpoT[Hs] or RelA[hS], respectively, to tune the HD versus SYNTH output ratio.

### SpoT_*Ab*_ is a monomer

It was shown earlier that Rel and RelA are prone to dimerization, a potential factor in their regulation^22,27–29^. This idea is subject of debate, with genetics^30^ and mass photometry^25^ suggesting that dimerization is unlikely to take place at physiological concentrations. We used small-angle X-ray scattering (SAXS) coupled to size-exclusion chromatography (SEC) to probe the conformation and oligomeric state of SpoT_*Ab*_ in solution (Fig. 2e,f).

SAXS–SEC data show that in solution SpoT_*Ab*_ has an oblate shape highly compatible with the crystal structure, with an *R*_G_ (radius of gyration) of 34.9 Å and roughly 85 kDa molecular weight estimated by SAXS (roughly 90 kDa by SEC) (Fig. 2e,f), in agreement with the 80 kDa theoretical molecular weight of monomeric SpoT_*Ab*_. The analysis of the normalized Kratky plot further supports a compact monomeric structure of SpoT_*Ab*_ in solution (Fig. 2f), and the ab initio SAXS envelope (Fig. 2g) is highly compatible with the τ-shaped X-ray structure of SpoT_*Ab*_. Collectively, these results demonstrate that in solution SpoT_*Ab*_ is a monomer in the τ-conformation.

### The inactive pseudo-SYNTH of SpoT_*Ab*_ is a regulatory domain

The pseudo-HD domain of RelA, evolved as a regulatory domain controlling catalysis via an intra-NTD allostery^31,32^. This is also the case with the HD specialization of SpoT_*Ab*_ where the pseudo-SYNTH domain has evolved into a strictly regulatory domain. Superposition of Rel_*Tt*_ SYNTH onto the pseudo-SYNTH of SpoT_*Ab*_ reveals extensive reorganization of the vestigial catalytic domain in SpoT_*Ab*_, consistent with differential conservation patterns in the G-loop and the ATP recognition motif (Supplementary Fig. 2a). These involve the residues that coordinate adenosine and guanosine (R249 to K240, R277 to E267 and Y329 to N304) and most phosphate-coordinating groups. Crucially, the catalytic residues D272 and Q347 are substituted for S263 and T321, respectively. These substitutions essentially impede the deprotonation and activation of the 3′-OH of GD(T)P, and Mg^2+^ binding, precluding the nucleophilic attack on the β-phosphate of ATP. We directly probed guanosine 5′-diphosphate (GDP) binding by SpoT_*Ab*_^NTD^ and RelA_*Ab*_^NTD^ by isothermal titration calorimetry (ITC). As expected, while SpoT_*Ab*_ does not bind GDP, RelA_*Ab*_ binds GDP with an affinity of 62 μM, which is similar to our earlier estimates for RelA_*Ec*_^NTD^ and Rel_*Bs*_^NTD^ (refs. ^25,31^) (Supplementary Fig. 2b,c).

### SpoT_*Ab*_ is not allosterically regulated by pppGpp

The synthetase activity of Rel/RelA is regulated via strong allosteric coupling between the HD and SYNTH domains that results in antagonistic conformational states^26,31,33^. Alarmones exploit this allosteric coupling to stimulate the SYNTH activity, however, this regulation is lost in SpoT_*Ec*_ (ref. ^31^). Our structure of SpoT_*Ab*_ provides a mechanistic interpretation. In the τ-state, the Core domain makes numerous contacts with SYNTH, providing further scaffolding to the already more stable version of SpoT_*Ab*_’s HD–pseudo-SYNTH hinge. Additional substitutions in the (p)ppGpp-allosteric site, Q203 (involved in ribose coordination and strictly conserved as A in RelA^31^) and in T209 (involved in phosphate coordination, typically K or R in RelA^31^), compromise alarmone-mediated regulation.

To directly validate the lack of pppGpp-mediated regulation in SpoT_*Ab*_, we characterized the interaction between pppGpp and SpoT_*Ab*_^NTD^ by ITC. As expected, SpoT_*Ab*_^NTD^ does not bind pppGpp allosterically (Supplementary Fig. 2d,e). Following the experimental approach used earlier for SpoT_*Ec*_^31^, we grafted the allosteric site of RelA_*Ab*_ (^236^RelA_*Ab*_^246^) onto SpoT_*Ab*_^NTD^ (replacing ^201^SpoT_*Ab*_^211^). As in the case of SpoT_*Ec*_, this resulted in a RelA-like affinity to pppGpp of the chimera RSH (*K*_D_ = 5.6 μM). These results support the generality of alarmone-mediated control being lost in SpoT and only present in SYNTH-active Rel/RelA stringent factors.

### The dipolar HD-active site is conserved between Rel and SpoT

**Fig. 3:**
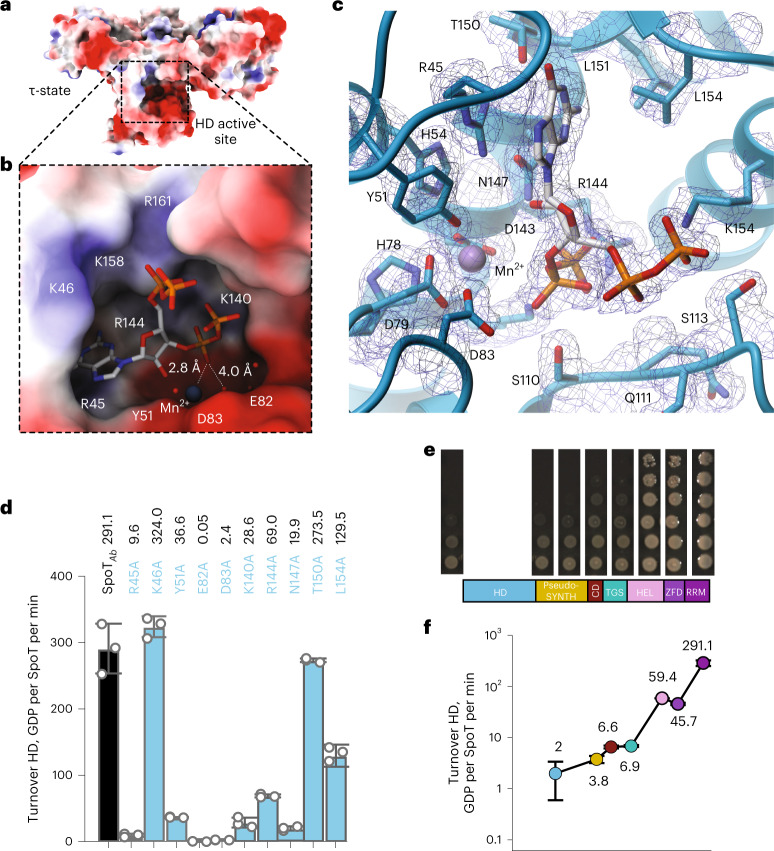
Defining features of *A. baumannii* SpoT catalysis. **a**, Surface representation of SpoT_*Ab*_ in the τ-state. The active site cavity in the HD domain is boxed in dashed lines. **b**, Zoom into the HD-active site of the SpoT_*Ab*_–ppGpp complex. The acidic half of the interface (residues R45, Y51, E82, D83 and K140) and the Mn^2+^ ion activate the water molecule for nucleophilic attack of the pyrophosphate bond pf ppGpp, while the basic half of the interface (K46, K158 and R161) stabilizes the 3′ and 5′ phosphates of the alarmone substrate. **c**, Ribbon representation of the active site of SpoT_*Ab*_ revealing the residues involved in coordination of ppGpp. The unbiased mFo-DFc electron density map corresponding to these residues is shown in dark blue. **d**, Effects of Ala substitutions in the ppGpp binding site on the HD activity of SpoT_*Ab*_. The residues for substitution were selected as per **c**. **e**, The HD functionality test of truncated versions of SpoT_*Ab*_. SpoT_*Ab*_ variants (each labeled on the figure) were coexpressed expressed with RelA_*Ab*_ in ∆*relA*∆*spoT* P*tac::relA A. baumannii* (AB5075). The ability of SpoT_*Ab*_ to promote the growth is reflective of its HD competence. **f**, HD activity of SpoT_*Ab*_ and the C-terminally truncated SpoT_*Ab*_ variants. Turnovers corresponding to each protein variant are colored as per the domain color code in **e**. Error bars represent s.d. of three or more independent samples examined over three independent experiments.

The electron density map of the SpoT_*Ab*_–ppGpp complex reveals that the alarmone is bound in high occupancy in all the SpoT_*Ab*_ molecules in the asymmetric unit (Supplementary Fig. 2f), with the coordination of the alarmone’s guanine base (Fig. 3a–c) resembling that in Rel_*Tt*_^NTD^-ppGpp^26^ and Rel_*Tt*_^NTD^-pppGpp^34^ complexes (Supplementary Fig. 3a,b). We probed the role of each residue involved in guanine coordination via Ala substitutions. While substitution of R45 (stacking the guanine) abrogated hydrolysis, removing van der Waals contacts to L154 decreased the activity approximately twofold: interactions with K46 were redundant (Fig. 3d). Disruption of the hydrogen bond of the guanine to T150 had only a minor effect. The additional hydrogen bond formed between the carbonyl group of the guanine and the enzyme’s backbone likely accounts for the guanine specificity of SpoT over adenosine.

The comparison of SpoT_*Ab*_–ppGpp with Rel_*Tt*_^NTD^–ppGpp^26^ reveals the shorter α6–α7 loop in SpoT_*Ab*_ as the main point of divergence (Supplementary Fig. 3a,b). This difference is consistent with the loss of allosteric regulation by pppGpp in SpoT_*Ab*_. As observed for Rel_*Tt*_^NTD^ (ref. ^26^), the HD-active site of SpoT_*Ab*_ displays a dipolar charge distribution with a basic half mediating the stabilization of the 5′- and 3′-polyphosphate groups of (p)ppGpp and the other highly acidic half mediating the 3′-pyrophosphate hydrolysis (Fig. 3a,b). Inspection of the complex reveals the crucial role of Y51 and the ^82^ED^83^ active site motifs as they work together with the Mn^2+^ cofactor to coordinate and stabilize a network of water molecules near the sugar-phosphate moiety during hydrolysis (Fig. 3b,c). Indeed, substitutions of Y51, E82, D83 or N147 render SpoT_*Ab*_ HD-inactive in our enzymatic assays (Fig. 3d). At the positively charged side, the 5′-polyphosphate is loosely coordinated and exposed to the bulk solvent. By contrast K140 and R144 hold the 3′-pyrophosphate in place and Ala substitutions of these residues decrease the activity of the enzyme between five- and tenfold suggesting these are key residues that orient the scissile bond.

### The CTD allosterically stimulates hydrolysis in the SpoT NTD

Until now, our understanding of the function of the CTD region of long RSHs was based exclusively on studies of Rel and RelA that highlighted the CTD role in the association with starved ribosomes resulting in the activation of SYNTH and the auto-inhibition of the factor’s SYNTH domain off the ribosome^19–22,25,35^. Weak HD activity of CTD-truncated Rels has also indicated a possible HD-stimulatory role through an intramolecular regulation of hydrolysis^25,36,37^, suggesting that a similar mechanism could also be at play in SpoT.

To probe this hypothesis, we characterized the HD activity—both in vitro and in vivo—of a set of C-terminally truncated variants of SpoT_*Ab*_ lacking (1) RRM (SpoT_*Ab*_^1–614^), (2) RRM and ZFD (SpoT_*Ab*_^1–560^), (3) RRM, ZFD and HEL (SpoT_*Ab*_^1–454^), (4) CTD altogether, that is, RRM, ZFD, HEL and TGS (SpoT_*Ab*_^1–385^), (5) CTD as well as the Core domain (SpoT_*Ab*_^1–339^) and, finally, (6) a variant consisting of just the HD domain (SpoT_*Ab*_^1–195^). These truncates were all generated at the endogenous *spoT* locus in a ∆*relA* P*tac::relA A. baumannii* strain, and the ability to grow on complex media supplemented with IPTG was evaluated as a proxy of (p)ppGpp hydrolysis.

While SpoT_*Ab*_ variants lacking the RRM or the RRM and ZFD domains retained the wildtype (WT) ability to sustain the bacterial growth—that is, could efficiently degrade (p)ppGpp synthesized by RelA—further C-terminal truncations compromised the in vivo HD functionality, as evidenced from pronounced growth defects (Fig. 3e). Biochemical assays agree with the in vivo data (Fig. 3f). Truncation of the RRM and ZFD decreases the HD activity fivefold. Further deletion of the TGS–HEL domains leads to a dramatic 42-fold decrease in activity. Truncations beyond the TGS compromised the activity by 70-fold or more and the isolated HD domain was nearly inactive. Collectively, our results indicate that the CTD region functions as an allosteric activator of the HD function of SpoT_*Ab*_.

### The Core domain is a linchpin that controls the τ-state

**Fig. 4:**
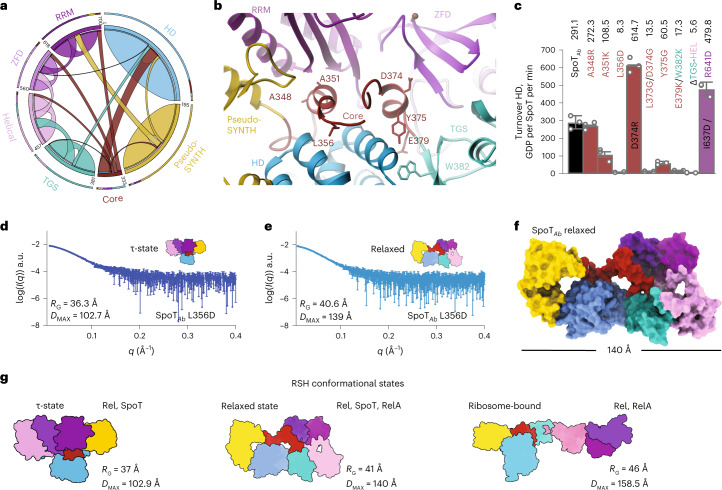
The CTD controls the hydrolysis activity of SpoT by controlling the equilibrium between HD-active τ-state and HD-inactive relaxed conformations. **a**, Circos plot of long-distance interactions (ten residues or more) within SpoT_*Ab*_. Each domain is defined and colored as in Fig. 2a. The thickness of connecting lines represent the number of contacts between two domains. **b**, Cartoon representation of the allosteric network defined by the Core domain connecting the domains of the enzyme in the τ-state. The key interface residues are shown as sticks and labeled. **c**, HD activity of crucial Core residues involved in interactions with other domains of SpoT_*Ab*_ (A384R contacting SYNTH, A351K contacting RRM, L356D contacting HD, L373G/D374G contacting ZFD, Y375G contacting the TGS). The TGS:HD interface is also probed with the E379K/W382K point mutant and ΔTGS–HEL versions. The τ-state stabilizing substitutions D374R and I637D/R641D increase the HD activity. **d**,**e**, SAXS curves of L356D in the τ-state (**d**) or relaxed state (**e**). **f**, Pseudo-atomic model of the relaxed state of SpoT_*Ab*_ calculated with Dadimodo^49^ using the experimental SAXS data from **e**. **g**, Cartoon representation of experimentally observed conformational states as well as particle dimensions of long-RSH enzymes. Error bars represent s.d. of three or more independent samples examined over three independent experiments.

Long-distance contacts in SpoT_*Ab*_ highlight the topology of an intradomain allosteric network (Fig. 4a), with the Core funneling information from the outer rim of the ‘cap’ to the HD-active site. To probe this, we introduced single point substitutions at each of the interfaces of the Core with the CTD (Fig. 4b) and measured the HD activity of these SpoT_*Ab*_ variants (Fig. 4c). An intact HD–Core–TGS interface—the structure involved in scaffolding the HD-active site—is crucial for hydrolysis, as the Y375G substitution resulted in a fivefold decrease in activity. While substitutions at the ZFD (L373G/D374G) and RRM (A351K) domain interfaces also resulted in pronounced defects (19- and threefold decreases, respectively), perturbations at the Core–pseudo-SYNTH domain interface (A348R) had only a minor effect. Finally, decoupling the contacts of the α6–α7 HD motif from the τ-cap via the Core L356D substitution, had a dramatic 35-fold decrease in hydrolysis, suggestive of an allosteric relay mediating a CTD-dependent activation of HD via the Core. We also observed that all these Core variants of SpoT_*Ab*_ had lower thermodynamic stability and loss of structure compared to the WT (Supplementary Fig. 4a,e and Supplementary Table 3). This indicates that an increase in the configurational entropy of the Core has a global effect in the dynamics and compactness of the enzyme.

We used SEC–SAXS to probe the role of each interface of the Core with the different domains of SpoT_*Ab*_ on the stabilization of the τ-state. The L356D substitution (SpoT_*Ab*_^L356D^, Fig. 4b and Supplementary Table 2) results in the segregation of the population into two conformational states with notable differences in *R*_G_ and particle dimensions (*D*_MAX_). In SpoT_*Ab*_^L356D^, one state is the τ-conformation observed in the crystal (Fig. 4d), while the other state is more relaxed (*R*_G_ = 41 Å, *D*_MAX_ = 130 Å) with dimensions reminiscent of the less compacted Rel and RelA—but not as elongated as in the ribosome-bound state (Fig. 4e). The pseudo-atomic model of this relaxed state calculated with Dadimodo^38^ indicates that the Core and HEL appear to have transitioned to a more disordered state that is consistent with the lack of structure of these regions in Rel/RelA when bound to ribosomes (Fig. 4f and Supplementary Fig. 5a); while the other domains retain their structural integrity. Prompted by this analogy, we next probed *A. baumannii* RelA_*Ab*_ and *B. subtilis* bifunctional Rel_*Bs*_ with SAXS. The dimensions of RelA_*Ab*_ (*R*_G_ = 42 Å, *D*_MAX_ = 130 Å, molecular weight of 88 kDa) are consistent with that of the relaxed state of SpoT_*Ab*_^L356D^ (Supplementary Fig. 5b and Supplementary Table 2), whereas Rel_*Bs*_ is populated by both relaxed and τ-states (Supplementary Fig. 5c,d and Supplementary Table 2). These SAXS results were further supported by chemical denaturation assays (Supplementary Fig. 5e,g and Supplementary Table 3). SpoT_*Ab*_ and Rel_*Bs*_ are substantially more stable than the monofunctional RelA_*Ab*_: the unfolding Gibbs free-energy of Δ*G*_u_^SpoT^ is 5.4 kcal mol^−1^, Δ*G*_u_^Rel^ is 4.6 kcal mol^−1^ and Δ*G*_u_^RelA^ is 2.2 kcal mol^−1^. The similar Δ*G*_u_ for Rel_*Bs*_ and SpoT_*Ab*_, together with high basal HD activity of Rel_*Bs*_ off the ribosome^25^, is consistent with Rel_*Bs*_ predominately sampling the HD-active compact and stable τ-state. Conversely, the relatively low stability of RelA_*Ab*_ is consistent with the enzyme predominantly populating the elongated STNTH-primed relaxed state.

Our results indicate that the Core conveys signals from the CTD to the HD as a function of the stability and compactness of SpoT_*Ab*_. The composition of the Core is the key to the conformational state of the enzyme as defined by the three main conformations observed in SpoT, Rel and RelA (Fig. 4g). Correlation of the decrease in HD activity, overall stability and loss of compactness on entropy-increasing substitutions such as A351K, L356D, L371G/D374G and Y375G supports the notion that an increase in structural disorder or flexibility of the Core (or the other IDRs) drives the conformational equilibrium away from the τ-state. The decrease in HD activity on destabilization of the τ-state is also consistent with suppression of (p)ppGpp hydrolysis in ribosome-bound τ-incompatible Rel^25^.

### The TGS domain acts as a scaffold for the HD-active site

**Fig. 5:**
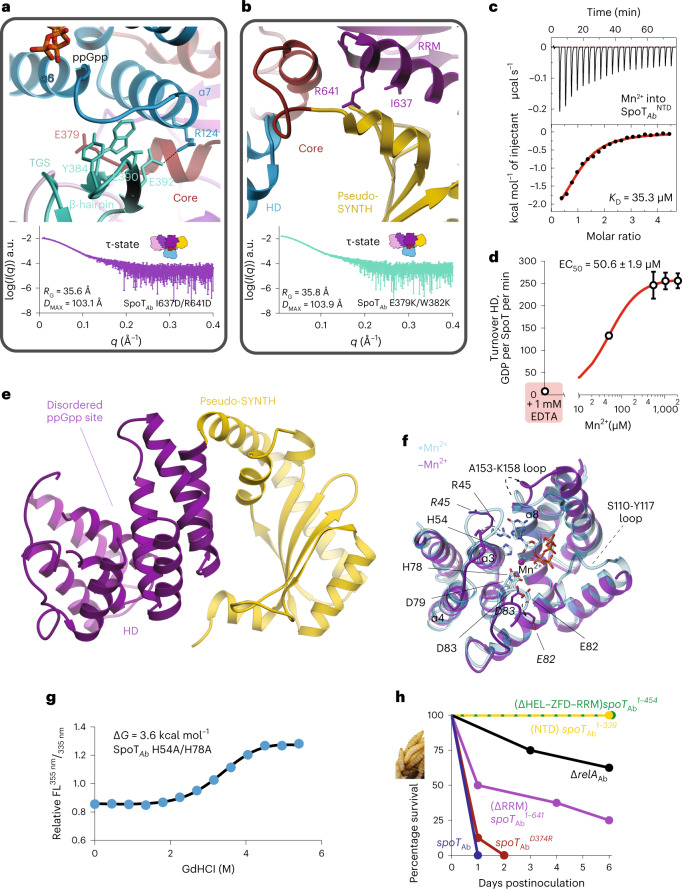
The Core domain of SpoT transduces the allosteric signal from the regulatory CTD and pseudo-SNTH to the enzymatic HD domain. **a**, Effects of substitutions at the α6–α7:Core–TGS interface. Interactions stabilizing the α6–α7 motif of the HD-active site with the Core wrapping around α7 and the TGS β-hairpin stabilizing α6. Experimental SAXS curve of SpoT_*Ab*_^E379K/W382K^ shows it remains in the τ-state. **b**, Effects of substitutions at the HD–Core–RRM interface with RRM locked in place via the Core and supporting interaction provided by pseudo-SYNTH, indeed the SAXS curve of SpoT_*Ab*_^I637D/R641D^ is consistent with the dimensions of the τ-state. **c**, ITC titration of Mn^2+^ into apo-SpoT_*Ab*_. **d**, HD activity of apo-SpoT_*Ab*_ as a function of increasing concentrations of Mn^2+^. **e**, Structure of the Mn^2+^-free SpoT_*Ab*_^NTD^. The HD domain is in purple, and the pseudo-SYNTH is in yellow. The disordered active site is labeled. **f**, Superposition of the HD domain of SpoT_*Ab*_ complexed with ppGpp (in light blue) onto Mn^2+^-free SpoT_*Ab*_ (in purple). The key differences in conformation of catalytically crucial active site residues and the structural elements α3, α4 and α8 are highlighted with dashed arrows and shown in bold, respectively. **g**, Thermal denaturation profile monitored by far UV circular dichroism (CD) spectrum at 222 nm of SpoT_*Ab*_^H54A/H78A^, which cannot bind Mn^2+^ in the HD domain. **h**, Virulence assays in the *G. mellonella* infection model demonstrate the essentiality of intact allosteric regulation of SpoT_*Ab*_ for virulence. *G. mellonella* larvae were injected with roughly 2 × 10 CFU of *A. baumannii* (AB5075) strains (10 µl at roughly 2 × 10^7^ CFU per ml), eight larvae were inoculated per strain and incubated at 37 °C in the dark. The viability of the larvae was scored every 24 h. Error bars represent s.d. of three or more independent samples examined over three independent experiments.

In Rel_*Tt*_, α6–α7 projects away from the HD catalytic center to accommodate the 3′ and 5′ polyphosphates and priming the enzyme for hydrolysis^26^. In SpoT_*Ab*_ the outward-pointing conformation of α6–α7 is further stabilized by the N-terminal region of TGS and Core domains that function as a clamp keeping α6–α7 in a hydrolysis-compatible position, with the HEL domain providing an additional support via the Core (Fig. 5a). The dramatic drop in the activity of a ΔTGS and ΔHEL SpoT_*Ab*_ variant (Fig. 4c) substantiates the functional importance of this stabilizing effect.

At the HD–TGS interface the β-hairpin of the TGS—involved in tRNA recognition in Rel^22,25,37^ and RelA^20,21,23^—is buried and stacks directly α6–α7 via a small hydrophobic interface formed by W382, Y384, L390 and the R124-E392 salt bridge (Fig. 5a). Disrupting this interface with the E379K/W382K substitutions (SpoT_*Ab*_^E379K/W382K^) led to a 17-fold decrease in the HD activity (Fig. 4a) suggesting that the HD:TGS interface constitutes an important allosteric signal transduction pathway. This scaffolding role is complemented by the Core that prevents the recoil of α6–α7 toward the HD-active site, which induces the opening of the NTD^26^. Despite the strongly attenuated HD activity of SpoT_*Ab*_^E379K/W382K^, SAXS showed SpoT_*Ab*_^E379K/W382K^ remains in the τ-state (*R*_G_ = 36 Å, *D*_MAX_ = 104 Å), suggesting an allosteric communication via the HD–Core–TGS axis (Fig. 5a and Supplementary Table 2).

Given that SpoT_*Ab*_ is SYNTH-inactive and is not specifically regulated by tRNA or ribosomes (Fig. 2d), it is not surprising that TGS residues involved in tRNA recognition—such as the crucial His residue involved in the recognition of the 3′ CCA end by Rel^22,25,37^ and RelA^20,23^—are lost in SpoT_*Ab*_ (S407 in SpoT_*Ab*_) but present in bifunctional SpoT_*Ec*_^1^. Moreover, the τ-state is sterically incompatible with the potential recognition of tRNA by TGS due to the sequestration the β-hairpin and α-helical elements. These observations suggest that SpoT_*Ab*_ TGS has been repurposed as a scaffolding domain that sustains hydrolysis by cooperating with the Core to lock α6-α7 in place to stabilize the HD-active site.

### ZFD and RRM domains fine-tune hydrolysis by SpoT_*Ab*_

With ZFD and RRM connecting with pseudo-SYNTH, the resulting interdomain interfaces are likely to play a role in the stability of the τ-state as well as to allosterically control HD via the HD–pseudo-SYNTH relay. Indeed, disruptive substitutions at the Core–HD (L356D), Core–pseudo-SYNTH–RRM (A351K) and Core–ZFD (L373G/D374G) that decreased the stability of the τ- state (Supplementary Fig. 3b–e) also decreased the HD activity by 35-, 3- and 22-fold, respectively (Fig. 4c). Therefore, we reasoned that substitutions stabilizing the Core–pseudo-SYNTH–RRM and Core–ZFD interfaces would, conversely, trigger an allosteric activation of hydrolysis (Fig. 5b).

To probe this, we introduced substitutions that would increase the contacts of RRM with pseudo-SYNTH via hydrogen bonds, I637D/R641D, and the Core with the ZFD, D374R (Fig. 5b). Denaturation experiments showed SpoT_*Ab*_^D374R^ and SpoT_*Ab*_^I637D/R641D^ have higher stability and compactness than the WT (Supplementary Fig. 6a-c and Supplementary Table 3) and SAXS measurements on SpoT_*Ab*_^I637D/R641D^ confirmed this variant retained the τ-state (Fig. 5b and Supplementary Table 2). As expected, the HD turnover of both variants increased (by 2.1- and 1.6-fold, respectively, Fig. 4c), and both behave like WT SpoT_*Ab*_ in vivo (Supplementary Fig. 6d).

Collectively, our results establish that HD activity is coupled to the stability of the τ-state: substitutions or interactions stabilizing the τ-state increase hydrolysis, whereas τ-state destabilizing substitutions lower the HD activity.

### Mn^2+^ ion organizes the HD-active site of SpoT_*Ab*_

The essential role of Mn^2+^ in (p)ppGpp hydrolysis is well documented for both Rel^25,33,39,40^ and SpoT_*Ec*_^41^. Our ITC measurements demonstrate that metal-free SpoT_*Ab*_^NTD^ binds Mn^2+^ with a *K*_D_ of 35.3 μM (Fig. 5c). Furthermore, while metal-free SpoT_*Ab*_ is completely HD-inactive, the HD activity is readily restored on addition of Mn^2+^ (Fig. 5d).

To directly reveal the structural role of Mn^2+^ we determined the X-ray structure of SpoT_*Ab*_^NTD^ in the metal-free state (Fig. 5e and Supplementary Table 1). Comparison with the SpoT_*Ab*_–ppGpp complex provides a structural explanation for the essentiality of Mn^2+^ in catalysis: in addition to its role in hydrolysis, by connecting α3, α4 and α8, Mn^2+^ coordination brings together the two halves of the HD domain and provides structural support to the active site (Fig. 5f). The removal of Mn^2+^ has a profound effect on the local conformation of the active site of SpoT_*Ab*_^NTD^. The catalytic ^78^HD^79^ and ^82^ED^83^ motifs are largely misaligned, loops S110-Y117 and A153-K158, involved in the 3′- and 5′-phosphate coordination are disordered, and the conformation of the T44–Y51 loop is incompatible with guanine coordination (Fig. 5f).

As expected from the lack of allosteric communication between HD and pseudo-SYNTH in SpoT_*Ab*_, these changes did not result in the opening of the enzyme’s NTD as observed in Rel_*Tt*_ on removal of Mn^2+^ (ref. ^26^). Thus, we used the stability of the enzyme as a proxy of the conformational state of a H54A/H78A-substituted variant of SpoT_*Ab*_ that cannot bind Mn^2+^. The decrease in conformational stability and compactness observed in SpoT_*Ab*_^H54A/H78A^ (Fig. 5g and Supplementary Table 3), suggests that the large perturbations to the active site induced by the absence of Mn^2+^ are sufficient to disrupt the τ-state and trigger a switch to the relaxed state (Supplementary Table 3).

Collectively, our results highlight the key role of Mn^2+^ in HD-catalysis and stabilization of the τ-state and suggest that as SpoT_*Ab*_ specialized, it lost the open/close allosteric switch between the HD and pseudo-SYNTH.

### Virulence of *A. baumannii* requires an intact τ-shaped SpoT_*Ab*_

(p)ppGpp-mediated signaling is crucial in antibiotic tolerance and virulence of *A. baumannii*^14,42^. We used the wax moth *Galleria mellonella* larvae infection model to assess the functionality of mutant *spoT*_Ab_ in supporting virulence of *A. baumannii* AB5075 (Fig. 5h). The *spoT*_Ab_^D374R^ strain killed 100% of the larvae within the first 2 days whereas 60% of the larvae survived 6 days of infection with the (p)ppGpp^0^ ∆*relA* strain. Infection with *A. baumannii* expressing the ΔRRM-truncated SpoT_*Ab*_^1–614^ resulted in 25% survival rate after 6 days. Notably, the RRM-truncated SpoT_*Ab*_^1–614^ had sixfold lower HD activity as compared to WT (Fig. 4c), and the strain displayed no growth defects when grown on Luria-Bertani plates (Fig. 3e). The defect in virulence becomes more prominent with truncations beyond the TGS domain: SpoT_*Ab*_^1–454^ and SpoT_*Ab*_^1–339^. The strong decrease in HD activity associated with the *A. baumannii* strains expressing these SpoT variants results in 100% larvae survival (Fig. 5h). Our infection assays demonstrate that while *A. baumannii* can tolerate the loss of several CTDs of SpoT without a dramatic fitness defect when grown in nonstressed laboratory conditions, fully functional intact intramolecular regulation of SpoT_*Ab*_ HD activity is crucial for a successful infection.

## Discussion

This study reveals the τ-state of full-length monofunctional SpoT_*Ab*_, which enables auto-stimulation of the HD activity by the CTD via the Core domain, to regulate (p)ppGpp hydrolysis. Together, Core, TGS and Mn^2+^, align the HD catalytic residues and stabilize the τ-state. Our observations indicate that compromising the functionality of any of these elements through substitutions of key residues results in large defects in hydrolysis. By contrast, pseudo-SYNTH, ZFD and RRM all subtly tune the HD activity of SpoT_*Ab*_ up or down by modulating its interactions with the Core. It is thus tempting to speculate that the loss of Mn^2+^-binding coupled to the monofunctionalization of RelA acted as the key structural change precluding the access to τ-state. Rel/RelA (p)ppGpp synthetases, lacking the Core are SYNTH-inactive and nonfunctional in vivo, with the minimal SYNTH-active version consisting of HD/pseudo-HD, SYNTH and Core domains^25,31,33,36^. Therefore, the presence of the Core and its crosstalk with the HD/pseudo-HD domain likely constitutes a universal structural requirement for the efficient stabilization of the active states of long RSHs.

**Fig. 6:**
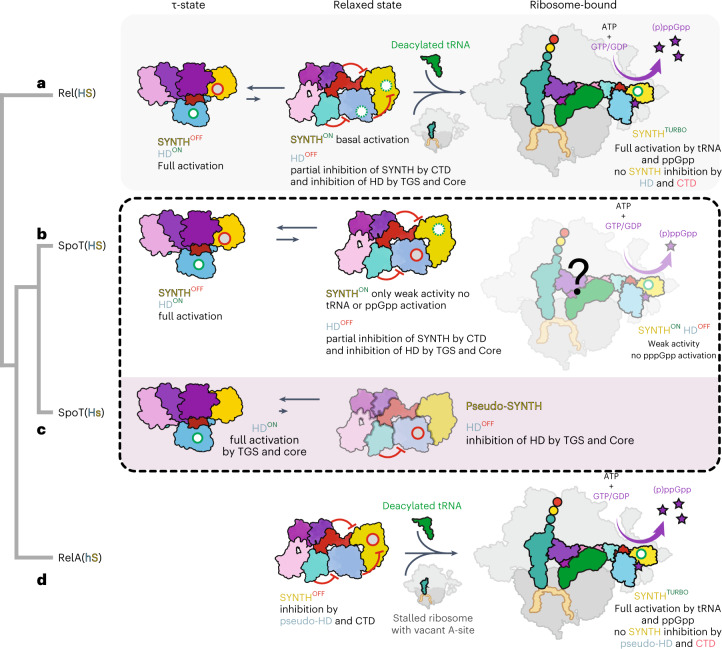
The enzymatic output of subfunctionalized RelA and SpoT RSH enzymes is evolutionarily tuned through constrains of the conformational landscape. **a**, Off ribosomes the ancestral bifunctional Rel[HS] assumes a τ-state with the CTD organizing the HD-active site and promoting the HD activity. This, in turn, strongly inhibits SYNTH activity via intra-NTD regulation. On amino acid starvation, Rel is recruited to starved ribosomal complexes. The ribosome-bound Rel assumes an extended conformation in which the auto-inhibitory effect of the CTD region on the SYNTH activity is released. The full activation of SYNTH is achieved on binding (p)ppGpp to an allosteric site within the NTD releasing the SYNTH inhibition by the HD domain. Thus, the full activation of either SYNTH or HD requires allosteric signaling from CTD to NTD enzymatic domains. **b**, Evolution of SpoT as a predominantly HD involved the loss of the allosteric control of the NTD by (p)ppGpp. In the bifunctional SpoT[HS] present in most Gamma- and Betaproteobacteria, the enzyme is capable of inefficient (p)ppGpp synthesis in the relaxed state despite the equilibrium strongly favoring the HD-active τ-state; it remains to be determined whether or not synthesis occurs on the ribosome. **c**, Subfunctionalization of SpoT in Moraxellaceae resulted in the monofunctional HD SpoT[Hs], which naturally populates only the compact τ-state, it is not control by pppGpp or starved ribosomes and is SYNTH-inactive. **d**, Subfunctionalization of Beta- and Gammaproteobacterial RelA[hS] constitutes the other extreme case of evolutionary restriction of the conformational dynamics of the ancestral Rel[HS]. While losing its HD activity, RelA retains all the allosteric elements of Rel involved in the regulation of (p)ppGpp synthesis and off the ribosome it does not assume the τ-state. Instead, it predominantly populates the functionally frustrated relaxed conformation, primed to switch to the elongated ribosome-associated state triggered by the 70S ribosome, uncharged tRNA and alarmones during stringency. Red circles represent inhibited catalytic centers, green circles represent fully activated catalytic centers and dashed green circles represent idling catalytic centers.

We propose a unifying scheme that rationalizes the evolution of the enzymatic output of long RSHs through finetuning of the conformational equilibrium of the τ-, relaxed and ribosome-bound states (Fig. 6). The presence of catalytically competent synthetase and HD domains in bifunctional Rel[HS] and SpoT[HS] requires both the τ and relaxed states as part of the conformational spectrum (Fig. 6a,b). While the τ-state primes Rel/SpoT for efficient (p)ppGpp hydrolysis, the less compact relaxed state sets the enzyme for low-efficiency (p)ppGpp synthesis. To fully activate its SYNTH activity, the enzyme needs further stimulation by starved ribosomes to attain the elongated state; this transition is possible for the amino acid starvation sensor Rel[HS] (Fig. 6a), but not for SpoT, which is not under allosteric control by starved ribosomes and (p)ppGpp^31^. In further subfunctionalized enzymes such as Moraxellaceae SpoT[Hs] and RelA[hS] the conformational landscape is limited compared to ancestral bifunctional Rel[HS] (Fig. 6c,d). Compared to SpoT[HS], in SpoT[Hs] the equilibrium is further shifted toward the τ-state required for hydrolysis (Fig. 6c). In contrast, in RelA[hS] the τ-state is inaccessible, and the enzyme is primed for ribosomal recruitment (Fig. 6d).

While full-length *A. baumannii* SpoT, *E. coli* RelA and *B. subtilis* Rel^25^, as well as *M. tuberculosis* Rel^40^ all behave as monomers in our hands, previous results indicate the possibility of long-RSH regulation via oligomerization^22,28,43^. However, the oligomerization model is largely supported by experiments with truncated protein variants, and these engineered RSH could potentially form contacts in *trans* that are naturally formed in *cis* in the full-length factor.

Expansion/contraction of disordered regions is the likely structural driver of the finetuning of catalysis in long RSHs through the restriction of the conformational space (Supplementary Fig. 1c,d). Longer IDRs and lack of Mn^2+^ binding favor the relaxed state in RelA[hS] and increase the frustration of the enzyme, whereas the shorter IDRs favor the compact HD-active τ-state in SpoT[Hs]. This genetic finetuning of catalysis, based on the optimization of the length and forces generated by IDRs, is reminiscent of the evolution of human glucocorticoid receptor isoforms^44^ or the UDP-α-d-glucose-6-dehydrogenase^45^. Such mechanisms seem to have evolved as a solution for conformationally heterogenous proteins with partially active resting states, that are under strong energetic and functional frustration.

The unifying scheme presented here provides a framework that can be used to rationalize the ‘hub’ nature of SpoT and how binding partners such as the acyl carrier protein and the regulator of RpoD could modulate its output^46,47^ or in the case of Rel/RelA how ribosomes prevent hydrolysis by exploiting this extensive allosteric network. For other stringent factors like Rel protein partners such EIIA^NTR^ and DarB^36,48^ could also modulate the intramolecular allosteric communication of the regulatory domains with HD by favoring of the τ- or relaxed states, thus conditioning the catalytic output of the enzyme.

## Methods

### Multiple sequence alignment

Sequences were aligned with MAFFT v.7.164b with the L-ins-i strategy^50^ and visualized with Jalview^51^.

### Construction of bacterial strains and plasmids

All strains and plasmids are listed in Supplementary Data. Plasmids for the expression of *A. baumanii* RelA and SpoT, were based on amplification from *A. baumanii* AB19606 cells with Q5 polymerase (NEB). The backbone of the pET28b plasmid was amplified using oligos R-pET-hisTEV-Bmt and F-pET-synt. The PCR products were cleaved *Bmt*I and *Eco*RI at 37 °C for 1 h, the plasmid backbone dephosphorylated with rSAP (NEB) for 30 min. The gene products were ligated at 20 °C, overnight and the mixture transformed into *E. coli* MC1061 strain and verified by sequencing.

For plasmids expressing SpoT_*Ab*_ variants (pET28b-SpoT_Ab_*), deletions and point mutations to *spoT*_Ab_ were introduced by amplifying the entire pET28b-SpoT_*Ab*_ plasmid with Q5 polymerase (NEB) using primer pairs (listed in Supplementary Data) of which one of the primers harbored the desired mutation. For the SpoT_*Ab*_^NTD^ or RelA_*Ab*_^NTD^ constructs, primers harboring a stop codon in the desired position were used. For the deletions of other domains of SpoT_*Ab*_, primers were flanking the region to be deleted. In all cases, the products were treated with *Dpn*I (NEB), purified through an Omega purification column, phosphorylated with polynucleotide kinase and ligated. The mixture was transformed into *E. coli* MC1061 strain and resulting plasmids verified by sequencing.

For pHR1186, an apramycin cassette was PCR amplified from pMHL2 with the primers 2213/2214 (Supplementary Data). The product was then digested with *Nco*I/*Bgl*II and ligated into the pK18mobsacB vector digested with the same enzymes.

To construct pHR1187 and pHR1188, upstream and downstream regions of *A. baumannii relA* (ABUW_3302) or *spoT* (ABUW_0309) were PCR amplified with the primers 2133/2134 and 2135/2136 or 2137/2138 and 2139/2140 (Supplementary Data) respectively. PCR products were then digested with *Xho*I/*Eco*RI (upstream region) and *Eco*RI/*Nhe*I (downstream region) and ligated into the pTOX5-Tet vector cut with *Xho*I and *Nhe*I. Then, fused upstream and downstream regions were PCR amplified with primers 2110/2188 (Supplementary Data) and ligated into the pHR1186 digested with *Sma*I.

For pHR1467, the downstream region of *spoT* was PCR amplified with primers 2139/2140. The product was digested with *Xba*I/*Nhe*I and ligated into pH1186 digested with the same enzymes.

To generate pHR1440, pHR1441, pHR1442, pHR1443, pHR1444 and pHR1445, a part of *spoT* CDS (ABUW_0309) was PCR amplified with the primers 2874/2875, 2876/2877, 2878/2879, 2880/2881, 2882/2883 and 2884/2885 (Supplementary Data), respectively, and *Xba*I digested. The products were then ligated into pHR1467 digested with *Sma*I/*Xba*I.

To generate pHR1446, pUC18-mini-Tn7-LAC backbone was PCR amplified with primers 2856/2857 and digested with *Avr*II. An *Avr*II/*Eco*RV fragment from pRCG-eGFP containing an apramycin cassette was then ligated to the previously *Avr*II digested PCR fragment.

For pHR1447, a part of pHR1446 was PCR amplified with the primers 2906/2907, digested with *Ase*I/*Sca*I and ligated into pHR1446 digested with *Nde*I/*Sca*I. A part of the intermediate plasmid was PCR amplified with the primers 2464/2908, digested with *Avr*II/*Bam*HI and ligated into the same plasmid digested with *Avr*II/*Bam*HI.

To generate pHR1448, pHR1447 backbone without its origin of replication was PCR amplified with primers 3100/3101 and digested with *Sca*I. The pBAD33 origin of replication was PCR amplified with the primers 3098/3099 and ligated to the previously *Sca*I digested PCR fragment.

To generate pHR1449, *A. baumannii relA* (ABUW_3302) CDS was PCR amplified with the primers 2867/2868 and digested with *Bam*HI/*Pst*I. The digested product was ligated into pHR1446 digested with the same enzymes.

To generate pHR1450 to pHR1459, *A. baumannii spoT* CDS were PCR amplified with the primers 2909/2910 on the corresponding pET28b plasmids and digested with *Nde*I/*Eco*RI. The digested PCR products were ligated into pH1447 digested with the same enzymes.

To construct pHR1460, pAGS1 backbone was PCR amplified with the primers 2417/2418 and digested with *Eco*RI. An *Eco*RI fragment from pRCG-eGFP containing an apramycin cassette was then ligated to the previously *Eco*RI digested PCR fragment. To generate pHR1461, *A. baumannii relA* (ABUW_3302) locus was PCR amplified with the primers 2472/2473, digested with *Avr*II/*Bgl*II and ligated into pHR1460 digested with the same enzymes. To generate pHR1462, a PCR fragment containing a tetracycline resistance cassette was PCR amplified with the primer 3000/3001, digested with *Avr*II/*Xho*I and ligated to pHR1461 digested with the same enzymes.

To generate the *A. baumannii* Δ*relA*, pHR1187 was introduced in the WT strain by natural transformation. Apramycin-resistant and sucrose-sensitive recombinant clones were first selected to launch an overnight culture in LB medium. In a second step, apramycin-sensitive and sucrose-resistant clones were screened by PCR using the primers 2141/2142 to identify Δ*relA* recombinant.

For the *A. baumannii* Δ*relA* Δ*spoT*, the same protocol was used by introducing the pHR1188 into the Δ*relA* strain and recombinants were screened with the primers 2143/2144.

pHR1440, pHR1441, pHR1442, pHR1443, pHR1444, pHR145 were introduced in the *A. baumannii* WT or Δ*relA* strains to generate *spoT1-454, spoT1-560*, Δ*relA-spoT1-196*, Δ*relA-spoT1-339*, Δ*relA-spoT1-385*, Δ*relA-spoT1-454*, Δ*relA-spoT1-560*, Δ*relA-spoT1-641* strains using the same protocol, and recombinants were screened with the primers 2143/2144.

To complement strains with the P*tac*::*relA* construct, pHR1449 and pTNS2 were cotransformed by natural transformation. To complement strains with the P*tac*::*spoT* construct and allelic variants, pHR1450 to pHR1459 and pTNS2 were cotransformed by natural transformation. Insertion downstream *glmS* at the attTn7 site was screened by PCR with the primers 2422/2466 on apramycin-resistant clones. To complement strains with the P*relA*::*relA* construct pHR1461 or pHR1462 were introduced by natural transformation.

### Growth assays

*E. coli* BW25113 cells were transformed with expression constructs either based on a high-copy IPTG-inducible vector pUC derivative pMG25 (pMG25::*relA* (ref. ^30^), pNDM220::*relA*^*ΔRRM*^, pNDM220::*spoR* or pNDM220::*spoT*^*ΔRRM*^) or on a low-copy IPTG-inducible vector, mini R1 plasmid pNDM220, which is present in 1–2 copies per chromosome^52^. For solid medium growth assays, tenfold serial dilutions of overnight LB cultures were spotted onto LB agar supplemented with 30 μg ml^−1^ ampicillin and 1 mM IPTG. For liquid medium growth assays, thousandfold dilutions of the overnight LB cultures were made in liquid LB supplemented with 30 μg ml^−1^ ampicillin and 1 mM IPTG, seeded on a 100-well honeycomb plate (Oy Growth Curves AB Ltd), and plates incubated in a Bioscreen C (Labsystems) at 37 °C with continuous medium shaking. Growth rates (μ_2_) were calculated as slopes of linear regression lines through log_2_-transformed optical density (OD_600_) data points.

### Virulence assays in *G. mellonella*

*G. mellonella* larvae (TruLarv) were purchased from Biosystems Technology. On reception larvae were stored at 17 °C. The day of the inoculation, larvae were incubated 1 h at 4 °C before injection. Overnight culture of *A. baumannii* were washed twice and diluted to a cell density of roughly 2 × 10^7^ CFU ml^−1^ in 0.9% NaCl, 10 µl were injected in the bottom left proleg of each larva. Eight larvae were inoculated per strain and incubated at 37 °C in the dark. Viability of the larvae was scored every 24 h for 6 days.

### Protein purification

For proteis production, pET28b plasmids with the different expression constructs were transformed to BL21(DE3) *E. coli* cells and grown overnight at 37 °C in LB media supplemented with kanamycin 50 µg ml^−1^, then diluted 500 times to fresh LB media (with kanamycin) and grown at 37 °C up to an OD_600_ 0.6. The temperature was lowered to 25 °C and protein expression induced with 0.5 mM IPTG, and collected after 5 h by centrifugation. Cells were resuspended in 50 mM Tris pH 8, 1 M KCl, 2 mM MgCl_2_, 1 mM TCEP, 0.002% mellitic acid supplemented with cOmplete protease inhibitor cocktail (Roche), flash frozen and stored at −80 °C until purification. Cells cracking was done with a French press and the soluble extract was separated by centrifugation and loaded on to a Cobalt gravity flow column equilibrated with buffer A (50 mM HEPES pH 8, 500 mM NaCl, 500 mM KCl, 10 mM MgCl_2_, 1 mM TCEP, 0.002% mellitic acid). The column was washed with 10 column volumes of buffer A and the protein was eluted with 4 column volumes of buffer B (buffer A containing 200 mM imidazole). The elution fraction was immediately transferred to a SEC column (GE Healthcare), equilibrated in the SEC buffer (50 mM HEPES pH 7.5, 500 mM NaCl, 500 mM KCl, 2 mM MgCl_2_, 1 mM TCEP, 0.002% mellitic acid (and 1 mM MnCl_2_ for all SpoT proteins)). Fractions containing the protein were concentrated to 1 mg ml^−1^ and the His-tag cleaved with tobacco etch virus (TEV) protease (1:100 molar ratio) at room temperature overnight. Tag-less protein was purified by SEC or in the case of SpoT_*Ab*_^NTD^, RelA_*Ab*_ and RelA_*Ab*_^NTD^ using Cobalt gravity flow and collecting the flow-through. The proteins were immediately used or flash frozen in liquid nitrogen and stored at −80 °C. Rel_*Bs*_ was purified as in ref. ^37^.

### Preparation of ^3^H-labeled ppGpp

Here, 250 nM *E. faecalis* RelQ was incubated in reaction buffer (10 mM MgCl_2_, 20 mM Tris-HCl pH 8.0, 100 mM NaCl) together with 2 mM ATP and 1 mM ^3^H-GDP (specific activity 80 cpm pmol^−1^, PerkinElmer) for 2 h at 37 °C to produce ^3^H-ppGpp. The mixture was loaded on anion-exchange column (MonoQ 5/50 GL; GE Healthcare), and nucleotides were resolved by a 0.5–2,000 mM LiCl gradient (with 0.5 mM EDTA, 2.5 mM Tris-HCl pH 8.0). Fractions containing ^3^H-ppGpp were pooled and precipitated by addition of potassium acetate to a final concentration of 0.3 M followed by addition of 2.5 volumes of ethanol. The suspension was incubated at −20 °C overnight and centrifuged (21,100*g*, 30 min, 4 °C). Resulting pellets were washed with absolute ethanol, dried, dissolved in 10 mM HEPES-KOH buffer (pH 8.2) and stored at −80 °C.

### Crystallization

Before crystallization SpoT_*Ab*_ and SpoT_*Ab*_^NTD^ were purified from the TEV-cleavage reaction in the SEC buffer and concentrated to 8–10 mg ml^−1^. Screening of crystallization conditions was carried out using the sitting-drop vapor-diffusion method. The drops were set up in Swiss (MRC) 96-well two-drop UVP sitting-drop plates using the Mosquito HTS system (TTP Labtech). Drops of 0.1 μl of protein and 0.1 μl of precipitant solution were equilibrated to 80 μl of precipitant solution in the reservoir. Commercially available screens LMB and SG1 (Molecular Dimensions) were used to test crystallization conditions. The conditions resulting in protein crystals (SG1, position G6 for Mn^2+^-free SpoT_*Ab*_^NTD^ and LMB screen position H7 for SpoT_*Ab*_) were repeated as 2 µl drops.

SpoT_*Ab*_ crystals were incubated mother liquor supplemented with 20 mM of ppGpp (Jena Bioscience) and 20% glycerol for cryo-protection. Soaking was performed at 4 °C to prevent hydrolysis of ppGpp at different times: 10 s, 20 s, 30 s, 1 min and 2 min before vitrifying in liquid N_2_. The best diffracting crystals, were those soaked between 20 and 30 s. The crystals of Mn^2+^-free SpoT_*Ab*_^NTD^ were soaked for a few seconds in a cryo-protection solution containing the mother liquor supplemented and 20% glycerol and directly vitrified in liquid N_2_.

X-ray diffraction data was collected at the SOLEIL synchrotron (Gif-sur-Yvette, Paris, France) on the Proxima 1 and Proxima 2A beamlines using an Eiger-X 16M detector. Because of the high anisotropic nature of the data, we performed anisotropic correction of the merged intensity data as implemented on the STARANISO server (http://staraniso.globalphasing.org/) using the DEBYE and STARANISO programs. In the case of the crystals of the SpoT_*Ab*_–ppGpp complex, the analysis of the data suggested a resolution of 2.51 Å (with 3.29 Å in a*, 2.75 Å in b* and 2.51 Å in c*). For Mn^2+^-free SpoT_*Ab*_^NTD^, the analysis of the data indicated a resolution of 2.79 Å (with 2.94 Å in a*, 2.94 Å in b* and 2.79 Å in c*).

### Structure determination

The data were processed using XDS and scaled with XSCALE or Aimless. In all cases, the unit-cell content was estimated with the program MATTHEW COEF from the CCP4 program suite^53^. Molecular replacement was performed with Phaser^54^. We used the coordinates of Rel_*Tt*_^NTD^ as search model for the NTD (Protein Data Bank (PDB) ID 6S2T)^26^ and the coordinates of *E. coli* RelA for the CTD^19–21^. Because of the intrinsic interdomain dynamics of Rel catalytic domains we search for a molecular replacement solution using each catalytic domain as an independent ensemble. The molecular replacement solution from Phaser was used in combination with Rosetta as implemented in the MR-Rosetta suit from the Phenix package^55^. After several iterations of manual building with Coot^56^ and maximum likelihood refinement as implemented in Buster/TNT^57^, the model was extended to cover all the residues (R/Rfree of 21.7/25.0%).

For the metal-free SpoT_*Ab*_^NTD^, we used the coordinates of the individual HD and SYN domains from the structure of SpoT_*Ab*_ as the search model for MR (olecular replacement) in Phaser. The structure was completed after several iterations of manual building with Coot^56^ and maximum likelihood refinement as implemented in Buster/Buster/TNT^57^ to an R/Rfree of 24.6/28.6%. Geometrical restraints of all small molecules were generated with the Grade Web Server (http://grade.globalphasing.org). Supplementary Table 1 details all the X-ray data collection and refinement statistics. In both cases, structural analysis was carried out using Chimera and Coot^56^ and long-distance interactions were plotted using Circos^58^.

### Analytical SEC

For analytical SEC, 100 µl of each protein at a concentration of 1 mg ml^−1^ was loaded on a Superdex 200 Increase 10300 column (GE Healthcare) equilibrated in the SEC buffer. The progress of the chromatography was monitored by the OD_280_.

### ^3^H-ppGpp hydrolysis assay

Mutational analysis of *A. baumannii* SpoT was carried out with reaction mixtures contained 50–400 nM SpoT, 200 μM ^3^H-ppGpp, 1 mM MnCl_2_, in HEPES:Polymix buffer, pH 7.5 (ref. ^37^) (5 mM Mg^2+^ final concentration). After preincubation at 37 °C for 2 min, the reaction was started by the addition of prewarmed SpoT and 5 μl aliquots were taken throughout the time course of the reaction and quenched with 4 μl of 30% formic acid supplemented with a cold nucleotide standard (4 mM GDP) for ultraviolet (UV) shadowing. Individual quenched timepoints were spotted polyethylenimine–thin-layer chromatography plates (Macherey-Nagel) and nucleotides were resolved in 0.5 M KH_2_PO_4_ pH 3.5 buffer. The thin-layer chromatography plates were dried, cut into sections as guided by UV-shadowing, and ^3^H radioactivity was quantified by scintillation counting in EcoLite Liquid Scintillation Cocktail (MP Biomedicals).

SpoT_*Ab*_ Michaelis constant (*K*_M_) and maximum reaction velocity (*V*_max_) of ^3^H-ppGpp hydrolysis were determined with 25–200 nM of SpoT_*Ab*_ and 10–600 μM ^3^H-ppGpp as a substrate in HEPES:Polymix buffer pH 7.5 (1 mM MnCl_2_, 5 mM Mg^2+^ final concentration). Reaction was started by adding prewarmed SpoT_*Ab*_. To test inhibition of SpoT_*Ab*_ (50 nM)^3^ of H-ppGpp hydrolysis (100 μM) we used 0.5–4.0 μM of *E. coli* ribosomes (70S), acetylated *E. coli* Val-tRNA^Val^ and deacetylated *E. coli* tRNA^Val^ (Chemical Block). The reaction was started by adding prewarmed SpoT_*Ab*_ to the substrate in HEPES:Polymix buffer. The effect of Mn^2+^ and EDTA on SpoT_*Ab*_ was tested with 50–2,000 μM of MnCl_2_ or 1 mM EDTA at the substrate concentration 100 μM of ^3^H-ppGpp. Reaction mixture in HEPES:Polymix buffer, pH 7.5 (5 mM Mg^2+^ final concentration) including EDTA or MnCl_2_ and 50–400 nM SpoT_*Ab*_ was preincubated at 37 °C for 2 min and the reaction was started by adding 100 μM (final concentration) of ^3^H-ppGpp. 5 μl aliquots were taken throughout the time course and ^3^H radioactivity was quantified as described above.

### Chemical denaturation assays

Chemical denaturation assays were done in 25 mM Tris pH 7.5, 500 mM NaCl, 500 mM KCl, 2 mM MgCl_2_, 1 mM MnCl_2_, 0.5 mM TCEP 0.002% mellitic acid supplemented different concentration of guanidinium hydrochloride)^59^. The final mixtures contained 0.2–0.3 mg ml^−1^ of protein and 0–5.4 M guanidinium hydrochloride. Denaturation was followed by tryptophan fluorescence emission spectra in a PTI fluorometer (Photon Technologies International). The samples were excited at 280 nm and denaturation was assayed by the ratio of fluorescence intensities at 355 and 335 nm. Samples were prepared in triplicate, incubated and measured at 37 °C. All data were analyzed with the CDpal package in ‘chemical denaturation’ mode.

### SAXS

For SAXS proteins were concentrated to 8 mg ml^−1^, flash frozen and stored at −80 °C. SAXS data were collected at the SWING beamlines (SOLEIL and ESRF synchrotrons, France) on a Pilatus 2 M detector using the standard beamline setup in SEC mode. Samples were prepared in 500 mM NaCl, 2 mM TCEP and 30 mM Tris-HCl pH 7.0. SEC–SAXS was done with a Shodex KW404-4F column coupled to an high-performance liquid chromatography system, in front of the SAXS data collection capillary. The sample flowed at 0.2 ml min^−1^ and the data collected at 10 °C. Radiation damaged frames were removed before data analysis. The data were analyzed with the ATSAS suite. SAXS-based models were derived from the coordinates of the SpoT_*Ab*_–ppGpp complex. The initial model was completed using Modeller. The calculation of ab initio shapes was done with the program DAMMIF from the ATSAS package. Supplementary Table 2 shows all the SAXS-derived parameters.

To model the structures of SpoT_*Ab*_ in the relaxed state, we used the program Dadimodo^38^ that refines multidomain protein structures against experimental SAXS data. The program was used via the webserver application (https://dadimodo.synchrotron-soleil.fr) defining the following rigid domains: body_1 1–107, 129–189, 801–801; body_2 201–239, 257–293, 307–333; body_3 380–443; body_4 455–512; body_5 572–605, 901–901, body_6 618–689 based on the linker regions observed in the structure of SpoT_*Ab*_. The quality of the SAXS-based models was assessed based on the metrics proposed by Rambo and Tainer^60^.

### ITC

Titrations were performed with an affinity ITC (TA Instruments) at 15 °C. GDP (Sigma Aldrich) stocks of 650–670 mM were diluted in 50 mM HEPES pH 7.5; 500 mM KCl; 500 mM; NaCl; 10 mM MgCl_2_; 1 mM TCEP; 0.002% mellitic acid, to a final concentration 1.0 mM. The purified SpoT_*Ab*_^NTD^ was concentrated by ultrafiltration (Amicon ultra, 0.5 ml 30 kDa, Merck Millipore) to 80–100 μM. All final concentrations were verified by the absorption using a Nanodrop One (Thermo Scientific). All ITC measurements were performed by titrating 2 µl of the nucleotide into the protein using a constant stirring rate of 75 rpm. Data were processed and analyzed using the NanoAnalyse and Origin suits.

### Reporting summary

Further information on research design is available in the Nature Research Reporting Summary linked to this article.

## Online content

Any methods, additional references, Nature Research reporting summaries, source data, extended data, supplementary information, acknowledgements, peer review information; details of author contributions and competing interests; and statements of data and code availability are available at 10.1038/s41589-022-01198-x.

## Supplementary information

Supplementary Information Supplementary Figs. 1–6 and Tables 1–4.

Reporting Summary

Supplementary Data 1 Oligonucleotides and bacterial strains.

## Data Availability

All coordinates are deposited in the PDB, accession numbers 7QPR and 7QPS. All data needed to evaluate the conclusions are present in the paper and/or the Methods. Additional data related to this paper may be requested from the authors.
