# Supplementary material for: Structure of SpoT reveals evolutionary tuning of catalysis via conformational constraint
Source: Nat Chem Biol. 2022 Dec 5;19(3):334–45. doi: 10.1038/s41589-022-01198-x (PMC9974481; doi:10.1038/s41589-022-01198-x)
Supplement: Supplementary file 1 — Supplementary Figs. 1–6 and Tables 1–4. [file 41589_2022_1198_MOESM1_ESM.pdf]

nature chemical biology

Article

https://doi.org/10.1038/s41589-022-01198-x

Structure of SpoT reveals evolutionary tuning of catalysis via conformational constraint

In the format provided by the authors and unedited

SUPPLEMENTARY FIGURES

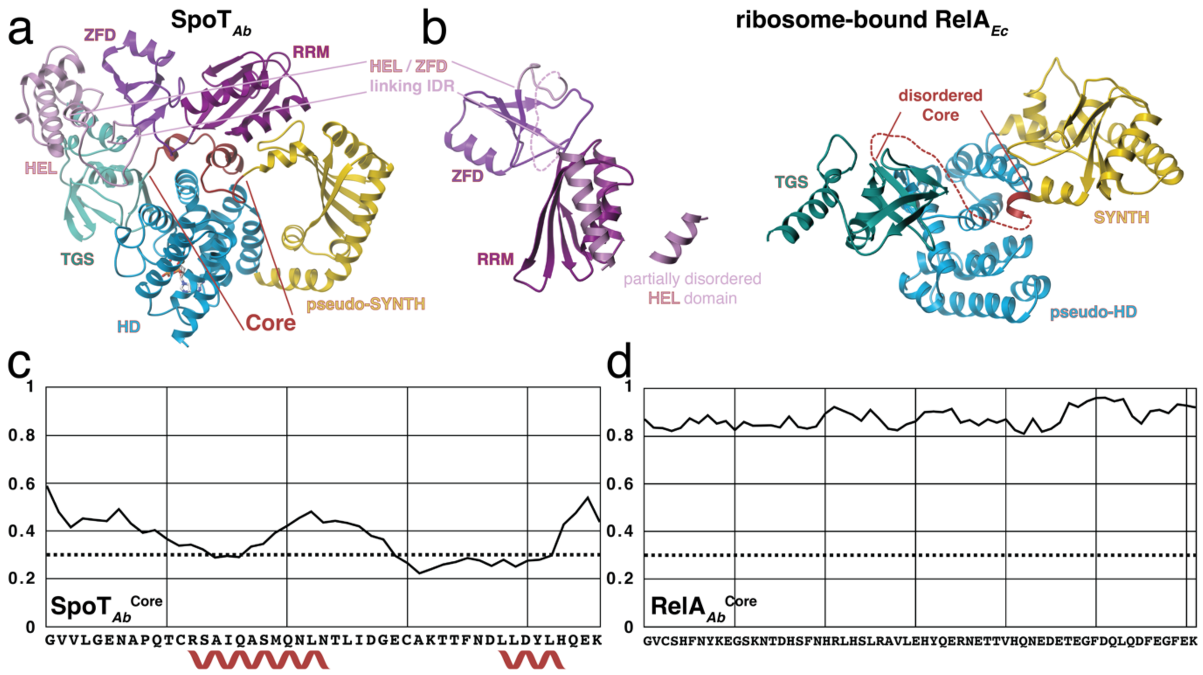

Supplementary Fig. 1. The linker regions connecting α-helices α6 and α7, SYNTH (pseudo-SYNTH) with TGS, and HEL with ZFD appear as highly flexible regions in the structures of free SpoT*Ab* as well as *E. coli* RelA bound starved ribosomal complex, related to Fig. 2. Cartoon representation of the structures of SpoT*Ab* (**a**) and *E. coli* RelA (Brown et al., 2016) in the conformation bound to 70S ribosomes and uncharged A-site tRNA (**b**). The intrinsically disordered linker regions that are not visible in the structures are highlighted in the figures and the individual domains are coloured as on Analysis of the intrinsic disorder propensity of the Core domain of SpoT*Ab* (**c**) vs RelA*Ab* (**d**) calculated with flDPnn. The two α-helices observed in the Core of SpoT*Ab* match the two stretches predicted by flDPnn with low disordered content. The comparison of both Core regions suggests RelA*Ab* Core is significantly more disordered than that of SpoT*Ab*.

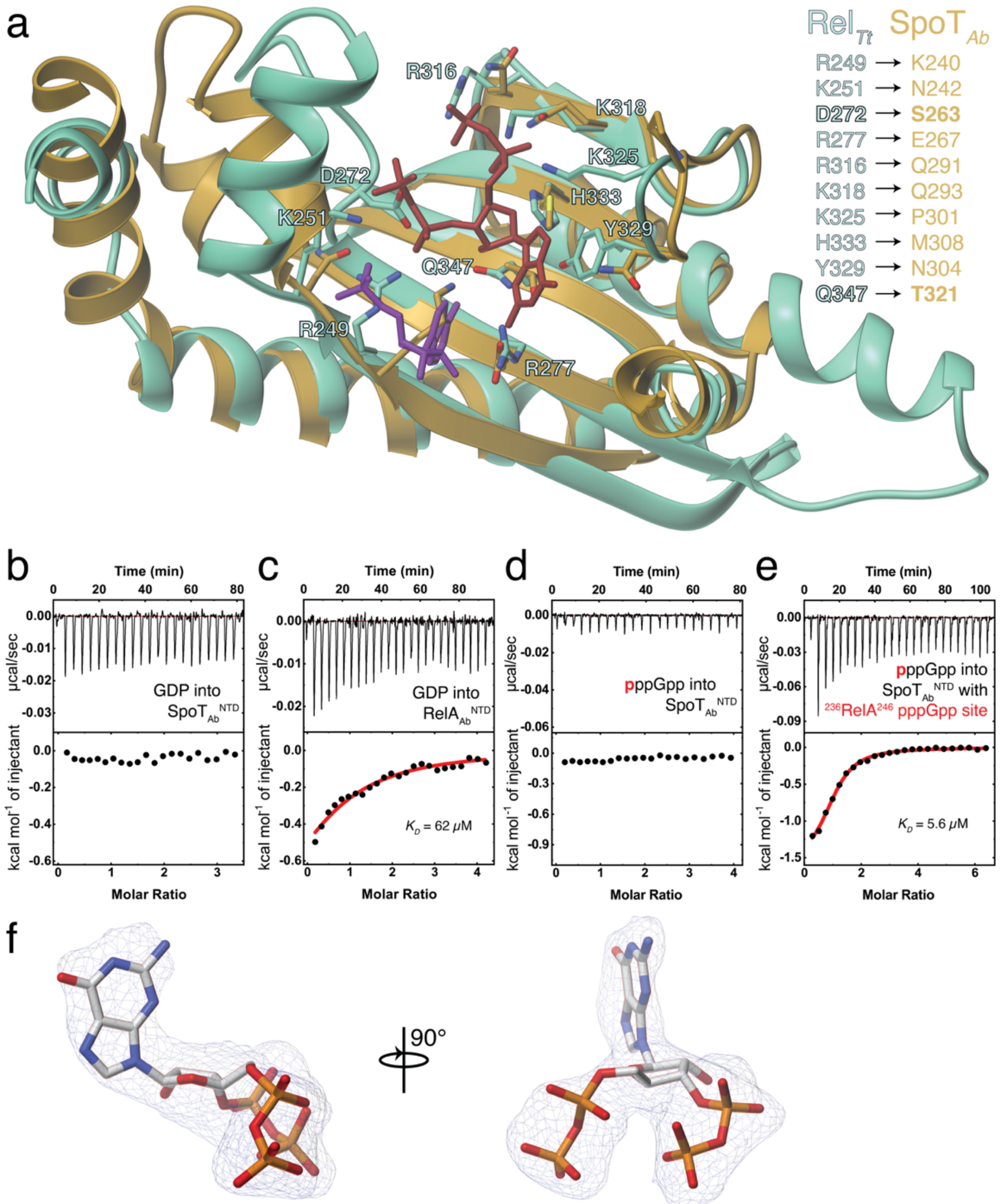

Supplementary Fig. 2. SpoT$_{Ab}$ is a monofunctional (p)ppGpp hydrolase with a degenerated pseudo-SYNTH domain, related to Fig. 2.
(**a**) Superposition of the pseudo-SYNTH domain of SpoT$_{Ab}$ (in gold) onto the SYNTH domain of *T. thermophilus* Rel (Rel$_{Tt}$) bound to ppGpp and AMP (in green). While the overall topology of the domain is conserved, a large number of substitutions in the nucleotide binding sites precludes substrate binding and catalysis of the pyrophosphate transfer. The crucial conserved Y that stacks with the guanine base of GDP and GTP is substituted to N, while the R residues that stacks with adenine (R249 and R277 in Rel$_{Tt}$) changed to K and E, respectively. Finally, catalytic residues D, E and Q residues involved Mg^{2+} coordination and hydrolysis changed to S263, 2319 and T321, respectively. ITC titrations of GDP into either SpoT$_{Ab}^{NTD}$ (**b**) or RelA$_{Ab}^{NTD}$ (**c**). ITC titration of pppGpp into SpoT$_{Ab}^{NTD}$ (**d**)and SpoT$_{Ab}^{NTD}$ with a grafted pppGpp-biding site of RelA$_{Ab}$, ^{236}RelA^{246} (**e**). In both cases the enzymes are supplemented with 1 mM APCPP, 1 mM GDP, and 10 mM EDTA. (**f**) Unbiased mFo-DFc electron density map of ppGpp bound to SpoT$_{Ab}$, after refinement with Buster/TNT and omitting ppGpp.

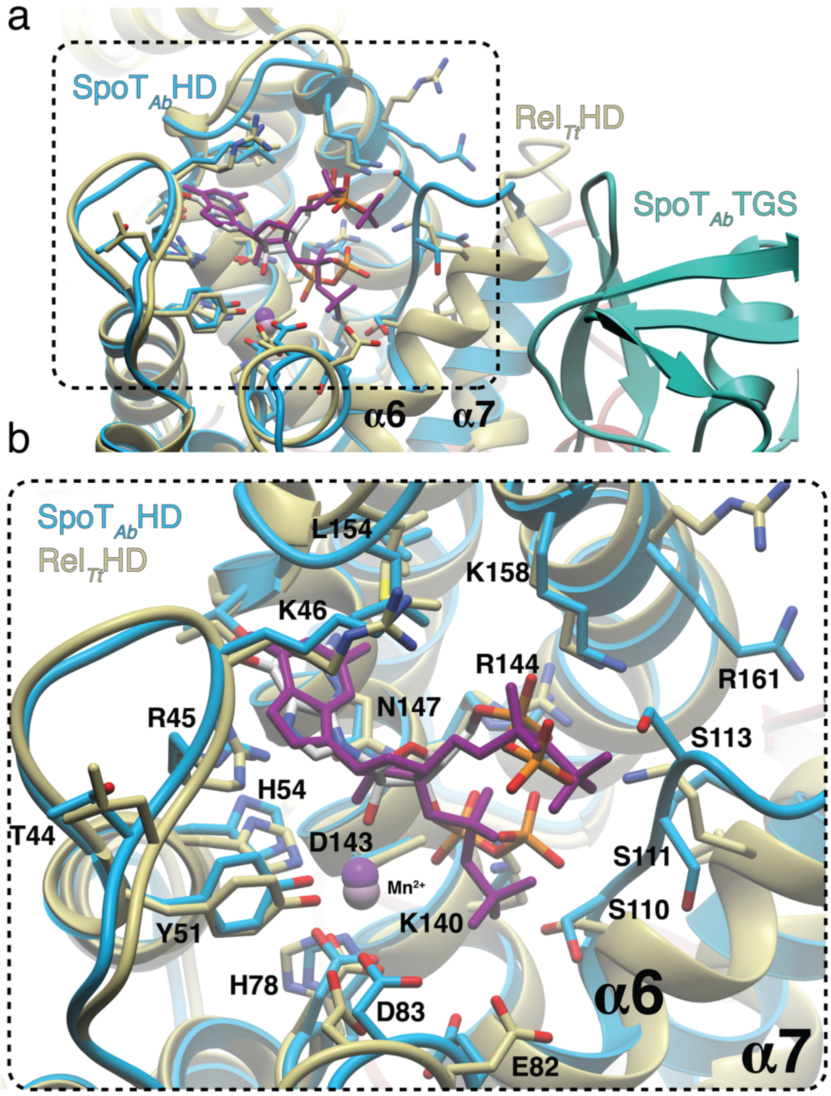

Supplementary Fig. 3. Conservation of the hydrolase active site architecture of SpoT$_{Ab}$ compared to Rel$_{Tt}$, related to Fig. 3. (**a**) Superposition of the HD domain of SpoT$_{Ab}$ bound to ppGpp (coloured as in **Figure 1a**) onto the HD domain of *T. thermophilus* Rel (Rel$_{Tt}$) bound to ppGpp (in yellow). From the superposition it becomes clear how the TGS domain of SpoT$_{Ab}$ keeps the shorter α6α7 loop locked into place. (**b**) Details of the strong conservation of the active site residues interacting with ppGpp. The only the region highly divergent between Rel$_{Tt}$ and SpoT$_{Ab}$ is α6α7 loop which in Rel$_{Tt}$ is part of the allosteric network that control the bifunctional activities of the enzyme, which is lost in SpoT$_{Ab}$.

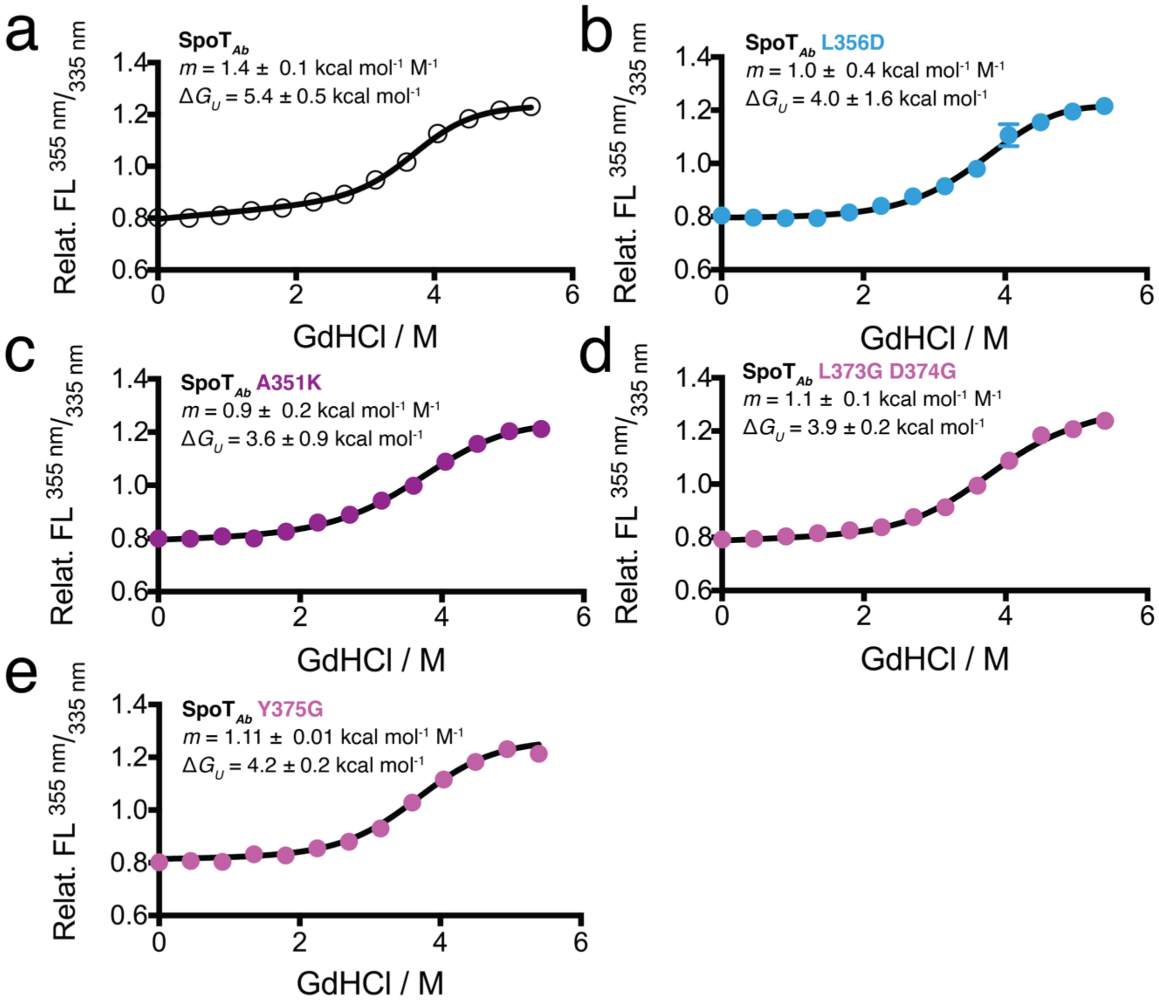

Supplementary Fig. 4. Effects of amino acid substitutions on the structural stability of SpoT*Ab*, related to Fig. 4. Thermal denaturation profiles monitored by far UV CD spectrum at 222 nm probing the α–helical content of wild-type SpoT*Ab* (**a**) as well as substituted variants (**b**-**e**).

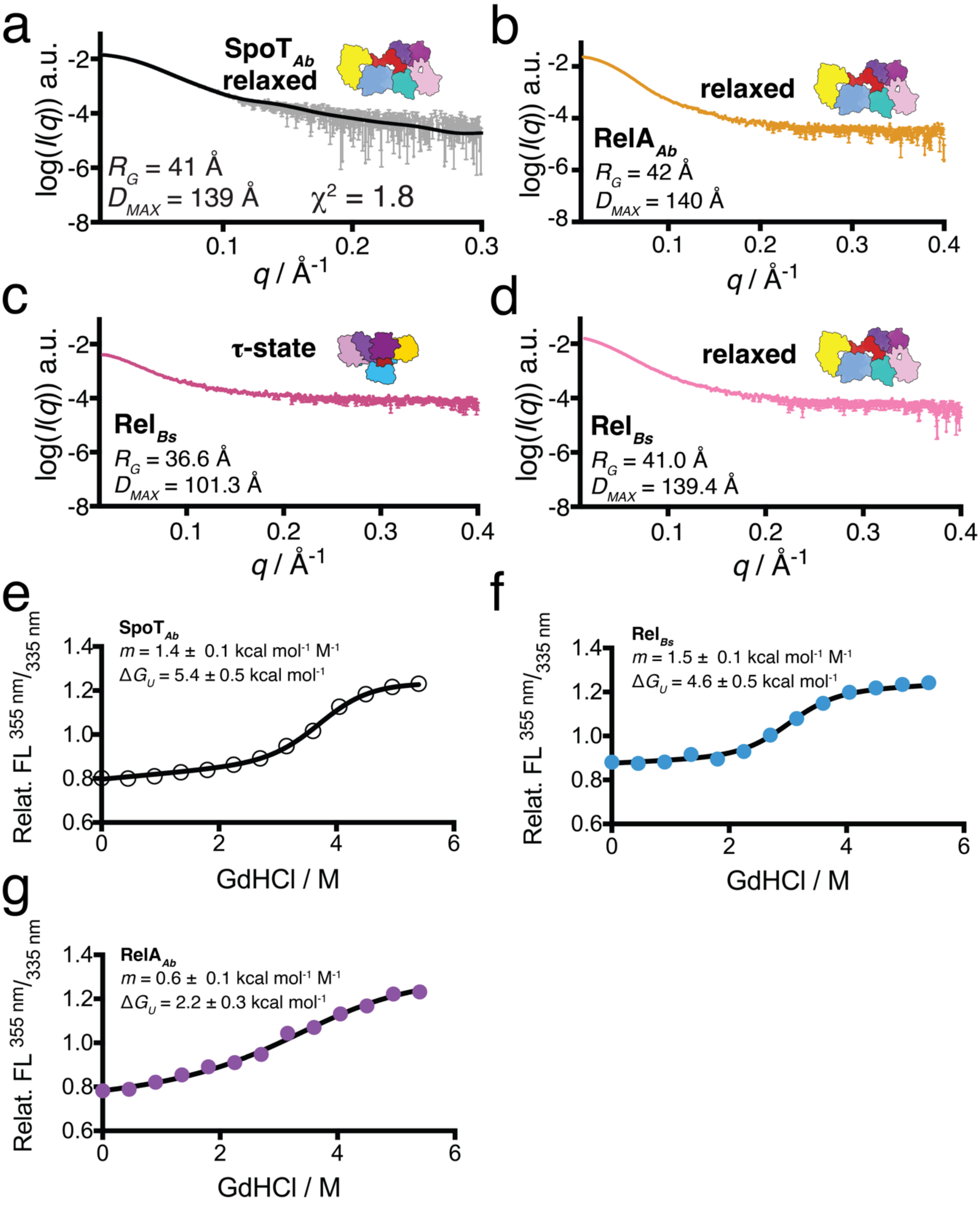

Supplementary Fig. 5. Effects of amino acid substitutions on the conformational state and structural stability of RSH enzymes (related to Fig. 4). (**a**) Comparison of the experimental SAXS data from the relaxed state of L356D (in grey) with the theoretical scattering curve of the relaxed state (solid line) obtained from the Dadimodo model. (**b**) SAXS curve of RelA$_{Ab}$ is consistent with the dimensions of the relaxed state. SAXS curves of Rel$_{Bs}$ in the τ-state (**c**) or relaxed state (**d**). Thermal denaturation profiles monitored by far UV CD spectrum at 222 nm of wild-type SpoT$_{Ab}$ (**e**) as a reference for a long RSH enzyme in a full τ-state, Rel$_{Bs}$ which contains both the τ- and relaxed state with the equilibrium predominately shifted to the τ-state (**f**), and RelA$_{Ab}$ which is fully in a relaxed state (**g**).

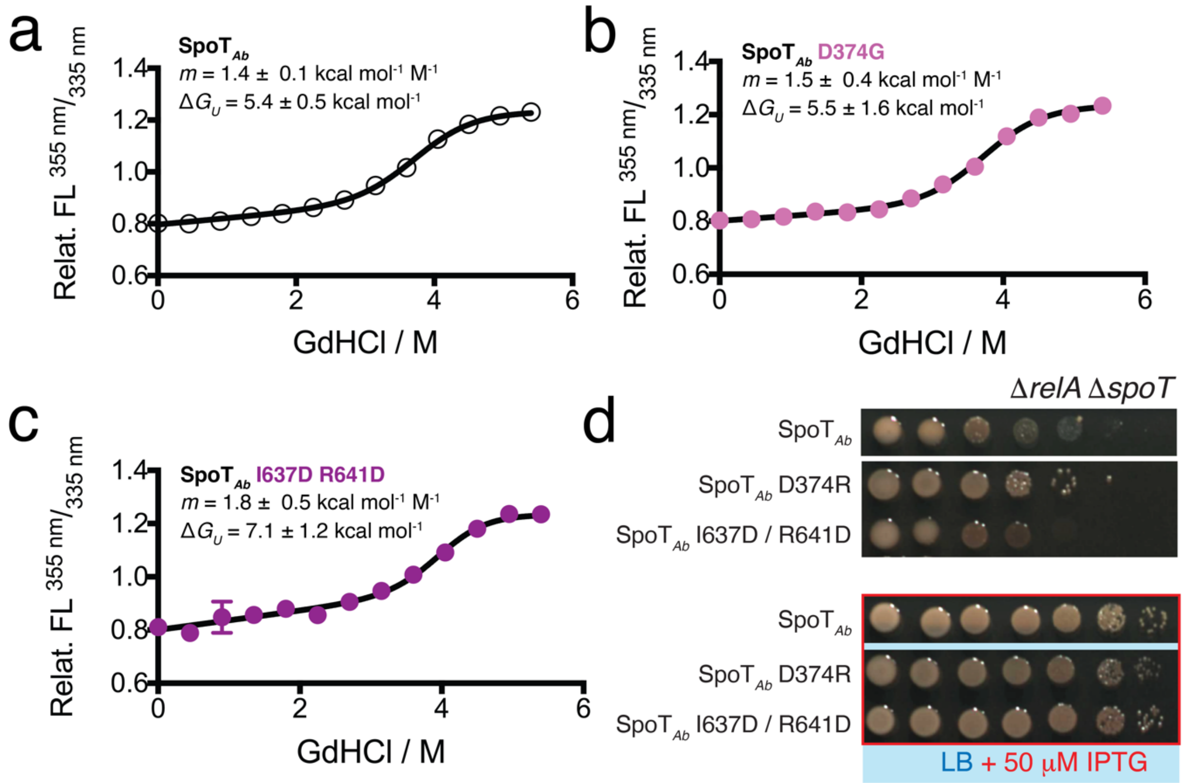

Supplementary Fig. 6. Effects of amino acid substitutions on the structural stability and *in vivo* activity of SpoT$_{Ab}$ (related to Fig. 5). Thermal denaturation profiles monitored by far UV CD spectrum at 222 nm probing the α–helical content of wild-type SpoT$_{Ab}$ (**a**) as well as substituted variants (**b** and **c**). (**d**) *In vivo* HD functionality tests of SpoT$_{Ab}^{D374G}$ and SpoT$_{Ab}^{I637D/R641D}$ expressed from the inducible P*tac* promoter in a Δ*relA*Δ*spoT* background of *A. baumannii* (AB5075) expressing *relA* from a replicative plasmid (pP*relA*::*relA*). The stabilising substitutions D374R and I637D/R641D phenocopy the WT.

SUPPLEMENTARY TABLES

Supplementary Table 1. X-ray data collection and processing.
The $CC_{1/2}$ criterion was used to determine the resolution range. Values for the outer shell are given in parentheses.

**Sample**	**SpoT$_{Ab}$-ppGpp complex**	**Mn^{2+}-free SpoT$_{Ab}^{NTD}$**
Diffraction source	Soleil PX1	Soleil PX2
Wavelength (Å)	0.9786	0.9801
Temperature (K)	100.0	100.0
Detector	Eiger-X 16M	Eiger-X 16M
Crystal-detector distance (mm)	402.12	254.68
Rotation range per image (°)	0.01	0.10
Exposure time per image (s)	0.01	0.004
Space group	$P2_12_12_1$	$P4_122$
a, b, c (Å)	128.8, 133.8, 211.3	90.9, 90.9, 262.4
α, β, γ (°)	90.0, 90.0, 90.0	90.0 90.0 90.0
Mosaicity (°)	0.20	0.20
Resolution range (Å)	48.88 – 2.51	85.90 – 2.79
Total N°. of reflections	1158971 (58614)	628934 (33155)
N°. of unique reflections	83804 (4191)	25329 (1230)
Completeness (ellipsoidal %)	95.5 (72.6)	95.6 (86.4)
Redundancy	13.8 (14.0)	24.8 (27.0)
$\langle I/\sigma(I)\rangle$	9.5 (1.5)	10.1 (2.0)
$CC_{1/2}$	0.995 (0.712)	0.990 (0.487)
R_{pim}	0.051 (0.459)	0.108 (0.689)
Overall B factor / Wilson plot (Å^2)	60.6	44.3
R-factor (%)	21.7	24.6
R_{free}-factor (%)	25.0	28.6
Ramachandran profile (%)		
Core	97.0	96.4
Allowed	3.0	3.6
Outliers	0.0	0.0
R.m.s. deviations		
Bond lengths (Å)	0.012	0.014
Bond angles (°)	1.56	1.55
Number of atoms	22600	5158
Macromolecules	21520	4893
Solvent	872	242
Other	208	23
B-factors (Å2)		
All atoms	69.9	49.4
Macromolecules	69.8	49.6
Solvent atoms	54.5	39.0
Other atoms	147.3	103.3
PDB ID	7QPR	7QPS

Supplementary Table 2. Average length of the different domains and interdomain regions of bifunctional Rel, and the monofunctional RelA and SpoT.

RSH	HD/pseudo-HD	α6/ α7 motif	pseudo-SYNTH /SYNTH	Core	TGS	HEL	HEL/ZFD IDR	ZFD	ZFD/RRM linker	RRM
RelA	167	24	130	56	86	83	33	48	12	76
Rel	166	11	144	52	77	87	25	45	12	74
SpoT	165	8	138	48	73	80	23	46	12	85

Supplementary Table 3. SAXS parameters.
Theoretically and experimentally determined SAXS parameters of the different protein species. The quality of the SAXS-based models was assessed based on the metrics proposed by Rambo and Tainer.

**Sample**	***Rg* (Å)**	***D_{max}* (Å)**	V_P (**$Å^3$**)	**V_C**	**Conformation**
$SpoT_{Ab}$	37.0	102.9	1.6	625	τ-state
$SpoT_{Ab}^{L356D}$	36.3	102.7	1.5	613	τ-state
$SpoT_{Ab}^{L356D}$	40.6	135.3	1.7	742	relaxed state
$SpoT_{Ab}^{E379K/W382K}$	35.8	103.9	1.4	607	τ-state
$SpoT_{Ab}^{I637D/R614D}$	35.6	103.1	1.3	583	τ-state
$RelA_{Ab}$	42.0	140.0	2.5	903	relaxed state
Rel_{Bs}	36.6	101.3	1.3	520	τ-state
Rel_{Bs}	41.0	139.4	1.8	696	relaxed state

Supplementary Table 4. Protein stability parameters as determined by chemical denaturation assays.

Experimentally determined thermodynamic parameters resulting from the denaturation of the different SpoT_{Ab} variants as well as Rel_{Bs} and RelA_{Ab}.

**Sample**	***m*** **(kcal mol^{-1} M^{-1})**	**ΔG$_u$ (kcal mol^{-1})**	**Conformation**
SpoT_{Ab}	1.4	5.4	τ-state
SpoT_{Ab}^{L356D}	1.0	4.0	relaxed state
SpoT_{Ab}^{A348R}	1.3	4.8	predominately τ-state
SpoT_{Ab}^{A351K}	0.9	3.6	relaxed state
$\text{SpoT}_{Ab}^{L373G/D374G}$	1.1	3.9	relaxed state
SpoT_{Ab}^{D374G}	1.5	5.5	τ-state
SpoT_{Ab}^{Y375G}	1.1	4.2	relaxed state
$\text{SpoT}_{Ab}^{I637D/R614D}$	1.8	7.1	τ-state
$\text{SpoT}_{Ab}^{H54A/H78A}$	1.0	3.6	relaxed state
Rel_{Bs}	1.5	4.6	predominately τ-state
RelA_{Ab}	0.6	2.2	relaxed state
